# Global miRNA expression profile reveals novel molecular players in aneurysmal subarachnoid haemorrhage

**DOI:** 10.1038/s41598-018-27078-w

**Published:** 2018-06-08

**Authors:** Katia de Paiva Lopes, Tatiana Vinasco-Sandoval, Ricardo Assunção Vialle, Fernando Mendes Paschoal, Vanessa Albuquerque P. Aviz Bastos, Edson Bor-Seng-Shu, Manoel Jacobsen Teixeira, Elizabeth Sumi Yamada, Pablo Pinto, Amanda Ferreira Vidal, Arthur Ribeiro-dos-Santos, Fabiano Moreira, Sidney Santos, Eric Homero Albuquerque Paschoal, Ândrea Ribeiro-dos-Santos

**Affiliations:** 1 Laboratório de Genética Humana e Médica, Programa de Pós-Graduação em Genética e Biologia Molecular, Universidade Federal do Pará, Belém, Brazil; 2 Programa de Pós-Graduação em Oncologia e Ciências Médicas, Núcleo de Pesquisas em Oncologia, Universidade Federal do Pará, Belém, Brazil; 3 Serviço de Neurocirurgia - Hospital Ophir Loyola, Unidade Neuromuscular do Complexo Hospitalar da UFPA, Belém, Brazil; 4 Serviço de Neurofisiologia Intraoperatória, Neurogenesis Instituto de Neurociências, Belém, Brazil; 5 Serviço de Neurocirurgia do Hospital das Clínicas da Faculdade de Medicina da USP, São Paulo, Brazil; 6 Laboratório de Neuropatologia Experimental, Universidade Federal do Pará, Belém, Brazil; 7 Grupo de Pesquisa Amazônia Neurovascular, Universidade Federal do Pará, Belém, Brazil

## Abstract

The molecular mechanisms behind aneurysmal subarachnoid haemorrhage (aSAH) are still poorly understood. Expression patterns of miRNAs may help elucidate the post-transcriptional gene expression in aSAH. Here, we evaluate the global miRNAs expression profile (miRnome) of patients with aSAH to identify potential biomarkers. We collected 33 peripheral blood samples (27 patients with cerebral aneurysm, collected 7 to 10 days after the haemorrhage, when usually is the cerebral vasospasm risk peak, and six controls). Then, were performed small RNA sequencing using an Illumina Next Generation Sequencing (NGS) platform. Differential expression analysis identified eight differentially expressed miRNAs. Among them, three were identified being up-regulated, and five down-regulated. *miR-486-5p* was the most abundant expressed and is associated with poor neurological admission status. *In silico* miRNA gene target prediction showed 148 genes associated with at least two differentially expressed miRNAs. Among these, *THBS1* and *VEGFA*, known to be related to thrombospondin and vascular endothelial growth factor. Moreover, *MYC* gene was found to be regulated by four miRNAs, suggesting an important role in aneurysmal subarachnoid haemorrhage. Additionally, 15 novel miRNAs were predicted being expressed only in aSAH, suggesting possible involvement in aneurysm pathogenesis. These findings may help the identification of novel biomarkers of clinical interest.

## Introduction

Subarachnoid haemorrhage (SAH) is caused by bleeding into the subarachnoid space^1,2^. When caused by a ruptured cerebral aneurysm, is called aneurysmal subarachnoid haemorrhage (aSAH). The aSAH presents high rates of mortality, ranging from 8% to 67%, and a significant morbidity for those who survive the initial haemorrhage^3,4^. Cerebral vasospasm (CV), also known as delayed cerebral ischemia (DCI), can be a severe complication of aSAH, contributing to unfavourable outcomes^2,5,6^.

Risk factors for aSAH include smoking, hypertension, female gender, alcohol intake, and positive family history of SAH, suggesting an active genetic component in the pathophysiology of this condition^7–10^. Several studies have found an association between aSAH risk and different genetic polymorphisms, such as in *eNOS*^11–13^. Nor prevention, neither risk assessment of aSAH has been proposed in the screening protocol of the patients. Therefore, efforts must be made to search for biomarkers capable of indicate the disease status, progression, and treatment responsiveness. Potential biomarkers may include DNA mutations, proteins, mRNA transcripts, and non-coding RNAs (ncRNAs).

Among the ncRNA, microRNAs (miRNAs) are expected to have potential application in the clinical setting. MiRNAs are small RNA molecules (19–25 nucleotides) with well-known biogenesis, which includes processing through the DGCR8/DROSHA, Dicer, Exportin-5, and RISC molecular complexes. Over ~2,500 miRNAs have been identified in humans, which may regulate more than 60% of protein-coding genes^14^. MiRNAs are characterized by variable expression in cells and tissues, which is influenced by the molecular cell environment. Differential expression of miRNAs has been shown associated with cancer-related diseases^15,16^, cardiovascular diseases^17,18^, and neurological conditions^19,20^. In addition, miRNAs are more resistant to degradation than mRNA and can be detected in fresh, fixed, or frozen tissue and peripheral blood samples. Distinctive patterns of circulating miRNAs have been identified for vascular diseases such as myocardial infarction, atherosclerotic disease and hypertension^21–24^. To date, few studies concerning miRNA expression profile in aSAH were published, even less using NGS techniques^25–29^. Compared to other technologies like qRT-PCR and Microarray, NGS provides high accuracy and sensitivity, and precise identification of miRNA sequences. One of the main advantages of NGS is the possibility of detection of known and novel miRNAs. Other advantages include the detection of miRNA expression levels in species without complete genome available and the use of barcodes during library preparation, allowing sample multiplexing^30^. Therefore, the purpose of the present study is to apply NGS to evaluate the global miRNA expression profile (miRnome) of patients with and without vasospasm after aSAH and verify potential biomarkers of this condition.

## Methods

### Sample collection

This study was reviewed and approved by the Ethical Committee of the Ophir Loyola’s Hospital (protocol number: 48199715.2.0000.5550). All study participants or their legal guardian provided informed written consent in accordance with the Helsinki Declaration of 1964. A total of 33 peripheral blood samples, collected at Ophir Loyola’s Hospital between 2014 and 2015, were included in the present study, comprising: (a) six control individuals with no evidence of cerebral aneurysm (verified by exams); (b) 14 patients with cerebral vasospasm after aSAH (Group 1); and (c) 13 patients without cerebral vasospasm after aSAH (Group 2). aSAH patient’s samples were collected 7 to 10 days after the haemorrhage, period when usually is the cerebral vasospasm risk peak^31,32^. All aSAH patients (n = 27) and controls (n = 6) were submitted to specific examination including computed tomography angiography and evaluated by neuroradiologist specialist. All participants in this study are from Brazil, a miscegenated population. Table 1 shows a summary of the participant characteristics (detailed information is available in Supplementary Material - Additional File 1).

**Table 1:** Clinical parameters of SAH groups and control participants.

	Group: Control (n = 6)	Group I: aSAH with cerebral vasospasm (n = 14)	Group II: aSAH without cerebral vasospasm (n = 13)
**Demographics**			
Age, years	50	52 ± 9	47 ± 11
% Female	67 (4)	86 (12)	77 (10)
**WFNS grade on admission, %**			
1–2	…	86 (12)	92 (12)
3–5	…	14 (2)	8 (1)
**CT feature on admission**			
% Fisher Grade 0, 1 or 2	…	29 (4)	85 (11)
% Fisher Grade 3 or 4	…	71 (10)	15 (2)
**Modifiable vasograde scale**			
Blue	…	0 (0)	8 (1)
Yellow	…	71 (10)	15 (2)
Green	…	29 (4)	77 (10)
Red	…	0 (0)	0 (0)

Note: The data are present in percentage (%) and the absolute number in parenthesis. WFNS indicates World Federation of Neurosurgical Societies, CT computed tomography and SAH, subarachnoid haemorrhage.

### Total RNA extraction and quantification

Peripheral blood samples (5 mL) were collected using Tempus Blood RNA Tube (Thermo Fisher Scientific, US) and stored at −20 °C until extraction. Total RNA was extracted using MagMAX RNA Isolation Kit (Thermo Fisher Scientific, US) and quantified with NanoDrop-1000 spectrophotometer (Thermo Fisher Scientific, US). Agilent RNA ScreenTape assay and 2200 TapeStation Instrument (Agilent Technologies, US) were used to detect and ensure RNA integrity.

### Small RNA library construction and sequencing

For small RNA-Seq, 1 (one) μg of total RNA per sample was used for library preparation using TruSeq Small RNA Sample Prep Kits (Illumina, San Diego, CA, USA). Size-distribution was measured with the DNA ScreenTape assay on a 2200 TapeStation system (Agilent Technologies, US) and a real-time PCR with KAPA Library Quantification Kit (Kapa Biosystem, US) was used to quantify and evaluate the quality of each library. A total library pool of 4 nM was sequenced using a MiSeq Reagent Kit v3 150 cycle on a MiSeq System (Illumina, San Diego, CA, USA).

### Small RNA-Seq *in silico* quantification

For the quantification of miRNAs expression levels after sequencing, three steps were followed:

#### Reads trimming and filtering

We used the Trimmomatic software, version 0.36^33^ to remove adaptors, low-quality bases and reads with less than 16 nucleotides. The parameter “ILLUMINACLIP TruSeq3-SE.fa:2:30:10” was used to remove read adaptors according to Illumina-specific sequences. “LEADING” and “TRAILING” parameters were set equal to 10 to cut the low-quality bases at the beginning or end of a read. A sliding window cut was applied to remove bases with average quality below 22 using the parameter “SLIDINGWINDOW:3:22”, and reads with less than 16 nucleotides were removed using “MINLEN:16”.

#### Aligning miRNA read sequences to the human genome

Reads were aligned using STAR, version 020201^34^, against the human reference genome (Hg19) obtained at UCSC database^35^. The parameters used include the maximum number of mismatch equal to 3 (“–outFilterMismatchNmax 3”); maximum intron length equal to 1 (“–alignIntronMax 1”); and the minimum number of bases matched to report an alignment was set higher than 16 nucleotides (“–outFilterMatchNmin 16”). The results files, generated as “.sam” by STAR, were manipulated with a set of utilities from Samtools^36^ and converted to “.bam” files. The “.bam” files contain the same information of the “.sam” files but in a binary conformation.

#### Measuring miRNA expression levels

For quantification of the expression of each miRNA, we used the HTSeq software, version 0.6.0^37^, using the human genome annotation file (“.gff”) and parameters set explicitly to miRNA and strand-specific modes.

### Statistical analysis

Data exploratory analysis was performed with R version 3.4.0^38^, RStudio and shell script. For storage and manage data, we use the relational database management system MySQL. MiRNAs that did not express in any sample (read count < 1) were excluded from downstream analysis. Differential expression analysis was performed using DESeq2 version 1.16.1^39^ and edgeR version 3.18.1^40^ according to their documentation. Two major parameters were considered in all differential expression analysis: adjusted *p*-value < 0.05 combined with |log2(foldchange)| > 1. Only miRNAs that met these criteria were considered differentially expressed (the complete list is available in Supplementary Material - Additional File 2). The statistical power analysis of the given sample sizes was evaluated using the R package RNASeqPower version 1.18.0^41^. Parameters were estimated considering only data from the differentially expressed miRNAs found. Sequencing coverage by miRNA was averaged between all 33 samples (27 cases + 6 controls) and the overall dispersion (coefficient of variation) was calculated using edgeR’s function “estimateCommonDisp”. Power was estimated for effect values of fold change 1.25, 1.5, 1.75 and 2, and False Discovery Rate’ alpha 0.1, 0.05, and 0.01 (Supplementary Table S1).

For other analyses (presented as boxplots and principal component analysis) the raw data count was normalised by counts per million (CPM). This normalisation is a measure of reads abundance used to compare the expression of miRNA in different samples or libraries sizes. CPM is calculated as follows:

$$CPM\,mapped\,reads=([\frac{row\,count}{total\,number\,of\,count\,in\,sample}]\times {10}^{6})$$

Wilcoxon signed-rank tests were performed to verify the statistical significance when comparing miRNA expression of patients against control.

### *In silico* identification of target genes and novel miRNAs

To identify potential target genes of the differentially expressed miRNAs found, we conducted an *in silico* analysis using three databases: (i) miRTarBase^42^, a curated database of miRNA target interactions; (ii) DIANA Tools with Tarbase version 7.0^43^, another manually curated target database; and (iii) miRTargetLink^44^, that offers detailed information on miRNA interactions in the form of interactive networks figures. All targets described by each database were stored in a local relational database to further manipulation of the information. For analysis, we considered only target genes with evidence of at least two distinct differentially expressed miRNAs (summed from Diana and miRTarBase).

Additionally, to take advantage of miRNA deep sequencing, we used the miRDeep2^45^ software to search potential novel miRNAs molecules. First, an index of the human genome reference (Hg19) was created with Bowtie1 (default parameters)^46^, then the script “mapper.pl” (available in miRDeep2 package) was used to map reads to the reference and collapse repeated sequences. After that, the script “miRDeep2.pl” was used to identify known and novel miRNAs using human’s mature miRNAs and pre-miRNA hairpins sequences, and species-related miRNAs from chimpanzee and mouse, obtained from the miRBase database (version 21)^47^. Only miRNAs with a score higher than five were considered for downstream analysis.

### Data availability

The raw sequencing reads of all libraries have been deposited at EBI-ENA (PRJEB24635).

## Results and Discussion

### Overview from small RNA-Seq

MiRNA sequence expression of 33 peripheral blood samples, comprising six control individuals and 27 aSAH patients, were analysed in order to identify potential biomarkers. From aSAH patients, 14 were associated with cerebral vasospasm (Group 1) and 13 without vasospasm (Group 2) (Table 1 and Supplementary Material - Additional File 1). Results section focus on the comparison of all aSAH patients against the control group. Result comparisons of specific subgroups (with and without vasospasm after an aneurysm) are available in Supplementary Material.

The average number of mapped reads per sample was 515,270, varying from 22,325 to 1,604,996. From a list of 2,576 known miRNAs, only 760 were found considered being expressed, with raw read count ≥1 in at least one sample. Considering the data from all samples (aSAH patients and controls), the most abundant miRNA detected was *hsa-miR-486-5p* (responsible for ~90% of the expression), followed by *hsa-miR-92-3p* and *hsa-miR-181a-5p*. Moreover, principal component analysis and hierarchical clustering of the Spearman correlation between the miRNA expression in each sample showed a clear contrast between patient and control samples (Supplementary Figs S1 and S2).

### Differential expression analysis

**Figure 1:**
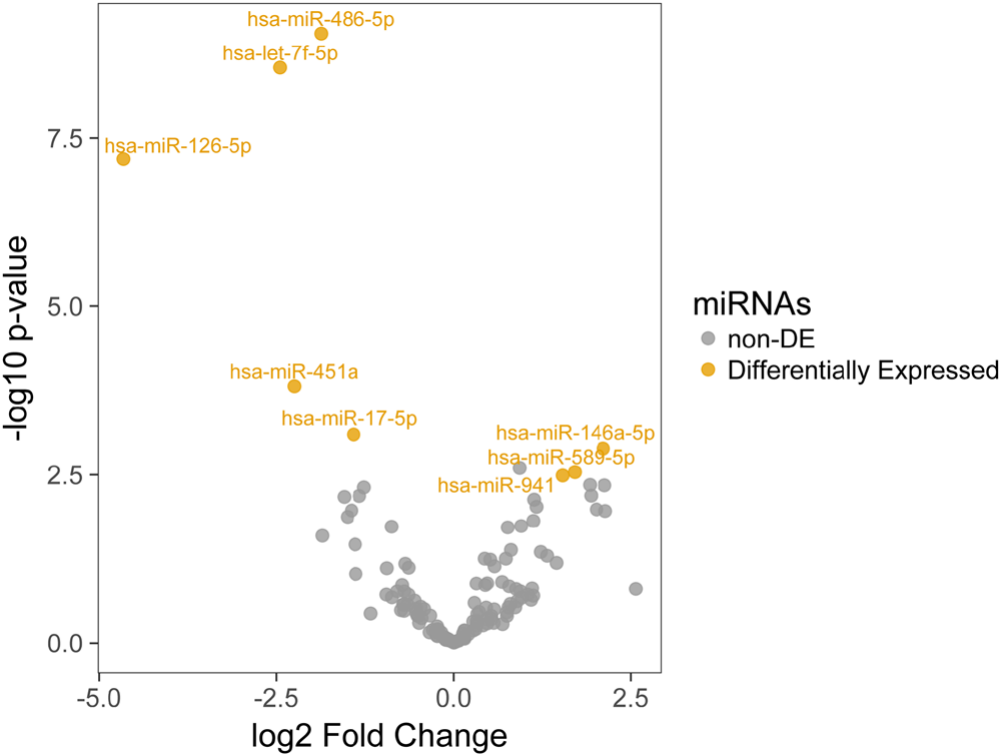
DE miRNAs between aSAH patients and control. The x-axis represents the values of log2(fold change) and y-axis are p-value in the scale of log10. Yellow dots are considered DE miRNAs under the conditions of adjusted p-value < 0.05 and |log2(fold change)| > 1. Grey dots are non-DE miRNAs. Note that, the three miRNAs on right of the figure are up-regulated, and five on the left are down-regulated.

Search for differentially expressed (DE) miRNAs between Group 1 (patients with cerebral vasospasm) and Group 2 (patients without vasospasm) showed no results according to the statistical significance threshold employed (adjusted p-value < 0.05 and |log2(fold change| > 1) (Supplementary Material - Additional Files 2 and 3). Comparing Group 1 and control, two DE miRNAs were found down-regulated: *let-7f-5p* and *hsa-miR-486-5p* (Supplementary Fig. S3). For Group 2 against control, three DE miRNAs were found*: let-7f-5p* and *hsa-miR-486-5p* (down-regulated) and *hsa-miR-146a-5p* (up-regulated) (Supplementary Fig. S4). Since no significant differences were found between aSAH Group 1 and Group 2, we compared control samples to all aSAH patients to enhance our statistical power. This analysis identified eight DE miRNAs, with: *let-7f-5p, hsa-miR-486-5p, hsa-miR-126-5p, hsa-miR-17-5p* and *hsa-miR-451a* being down-regulated and *hsa-miR-146a-5p*, *hsa-miR-589-5p*, and *hsa-miR-941* being up-regulated in aSAH patients. Figure 1 and Table 2 presents the statistical analysis results from DESeq2. Using EdgeR, a higher number of miRNAs could be identified being DE (Supplementary Table S2). However, methods showed overlapping results, with the same eight miRNAs being indicated by both (DEseq2 and EdgeR), supporting the results obtained.

**Table 2:** DE miRNAs between aSAH patients and control individuals.

miRNA	log2 (Fold Change)	p-value	Adjusted p-value	Expression in aSAH (vs control)
*let-7f-5p*	−2.447110513	2.86E-09	1.84E-07	Down-regulated
*hsa-miR-126-5p*	−4.657357337	6.49E-08	2.79E-06	Down-regulated
*hsa-miR-146a-5p*	2.109105434	0.00130016	0.027953449	Up-regulated
*hsa-miR-17-5p*	−1.409648419	0.000805205	0.0207743	Down-regulated
*hsa-miR-451a*	−2.245256235	0.000153327	0.0049448	Down-regulated
*hsa-miR-486-5p*	−1.868114599	8.91E-10	1.15E-07	Down-regulated
*hsa-miR-589-5p*	1.705526799	0.002900665	0.046536044	Up-regulated
*hsa-miR-941*	1.532605949	0.003246701	0.046536044	Up-regulated

**Figure 2:**
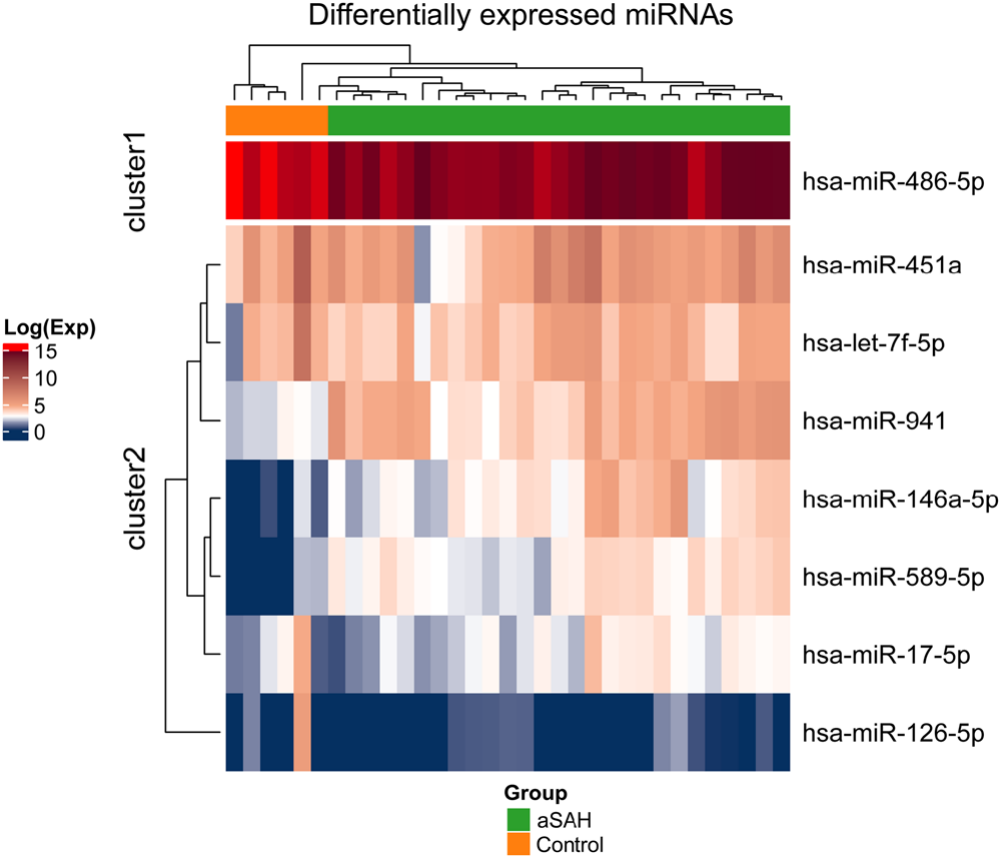
Heatmap and a hierarchical clustering of eight DE miRNAs. Green colour in top bar refers to aSAH patients and orange colour represents control individuals. In the Heatmap, dark-blue colour corresponds to lower expression, and dark-red colour corresponds to high expression in log scale.

Analysis of DE miRNAs expression data showed differences between expression levels of miRNAs among aSAH patients and controls (Fig. 2). Also, is possible to notice the elevated relative expression of *hsa-miR-486-5p* in both groups (~90% of all expression). The miRNA *hsa-miR-486-5p* (down-regulated in our results) has been described as one of the most down-regulated miRNAs in lung tumour and contributing to cancer progression by regulating Rho GTPase-activating protein^48^. This miRNA was described being differentially expressed in patients with gastric adenocarcinoma compared with healthy control^49^, and its deregulation was found as being a common event in both benign and malignant breast tumours^50^. In aneurysmal subarachnoid, the *hsa-miR-486–5p* was described in patients with a poor neurological admission status (WFNS score 4–5) compared with those with a good status (WFNS score 1–3)^51^. Additionally, another down-regulated miRNA found, *hsa-miR-451a*, commonly used in panels to evaluate haemolysis, has been related to the rupture or destruction of red blood cells^51^, which may indicate a relationship with aSAH clinical outcomes.

Other miRNAs listed in this study as differentially expressed also has been found in related studies of stroke, subarachnoid haemorrhage or cerebral ischemia, providing additional support to our results and offering insights about the role of these miRNAs. In brief, we comment and discuss each one below.

The *hsa-let-7f-5p*, whose nomenclature was maintained without “miR”, for historical reasons due to the first description of the Let-7 miRNA precursor in a developmental timing study in *Caenorhabditis elegans*^52^, and later found conserved from worms to humans, was observed here being down-regulated in aSAH patients. The *hsa-let-7f-5p* was also found significantly down-regulated in stroke cases^53^ and showing similar expression among various stroke samples^54^.

Another DE miRNA found being down-regulated in aSAH, was the *hsa-miR-126-5p*. The miR-126 family is expressed in endothelial cells and modulates angiogenesis *in vivo*^55^. Targeted deletion of miR-126 in mice has shown to result in vascular leakage and hemorraging^56^. This miRNA also has been described as down-regulated in different human stroke cases^53^, validated by stem-loop real-time PCR in young stroke patients (aged between 18–49 years)^54^ and differentially expressed in circulating blood of patients after stroke^29^.

The *hsa-miR-146a-5p* (up-regulated in aSAH patients), is involved in the regulation of inflammation and regulation of innate immune responses in monocytes and macrophages^57^. Taganov *et al*. screened for up-regulated miRNAs in monocytic cell line and showed that miR-146a and miR-146b unveiled a pattern of induction in response to a variety of microbial components and proinflammatory cytokines^58^. Yamasaki *et al*. in a study with 15 patients with osteoarthritis concluded that miR-146 is intensely expressed and might play a role in osteoarthritis cartilage pathogenesis^59^. In the same way, *hsa-miR-146a* has been found up-regulated in brain tissues, that suppress expression of COX-2 in neurological disorders^60^. Bache *et al*. reported a relative increase in *hsa-miR-146a-5p* expression in cerebral fluid from SAH patients with Delayed cerebral ischemia (DCI), compared with those without DCI (P < 0.05)^51^.

The *hsa-miR-17-5p* appears to be closely linked to the early evolution of the vertebrate lineage^61^. However, as miRNAs *hsa-let-7f-5p* and *hsa-miR-126-5p*, the *hsa-miR-17-5p* was listed as differentially expressed in patients after stroke in a study of Tiedt *et al*.^29^ and it was one of the 66 miRNAs that exhibited an increase expression in cerebral fluid from SAH patients when compared to neurologically healthy control (p < 0.001) – Supplemental Table II from Bache *et al*.^51^.

**Figure 3:**
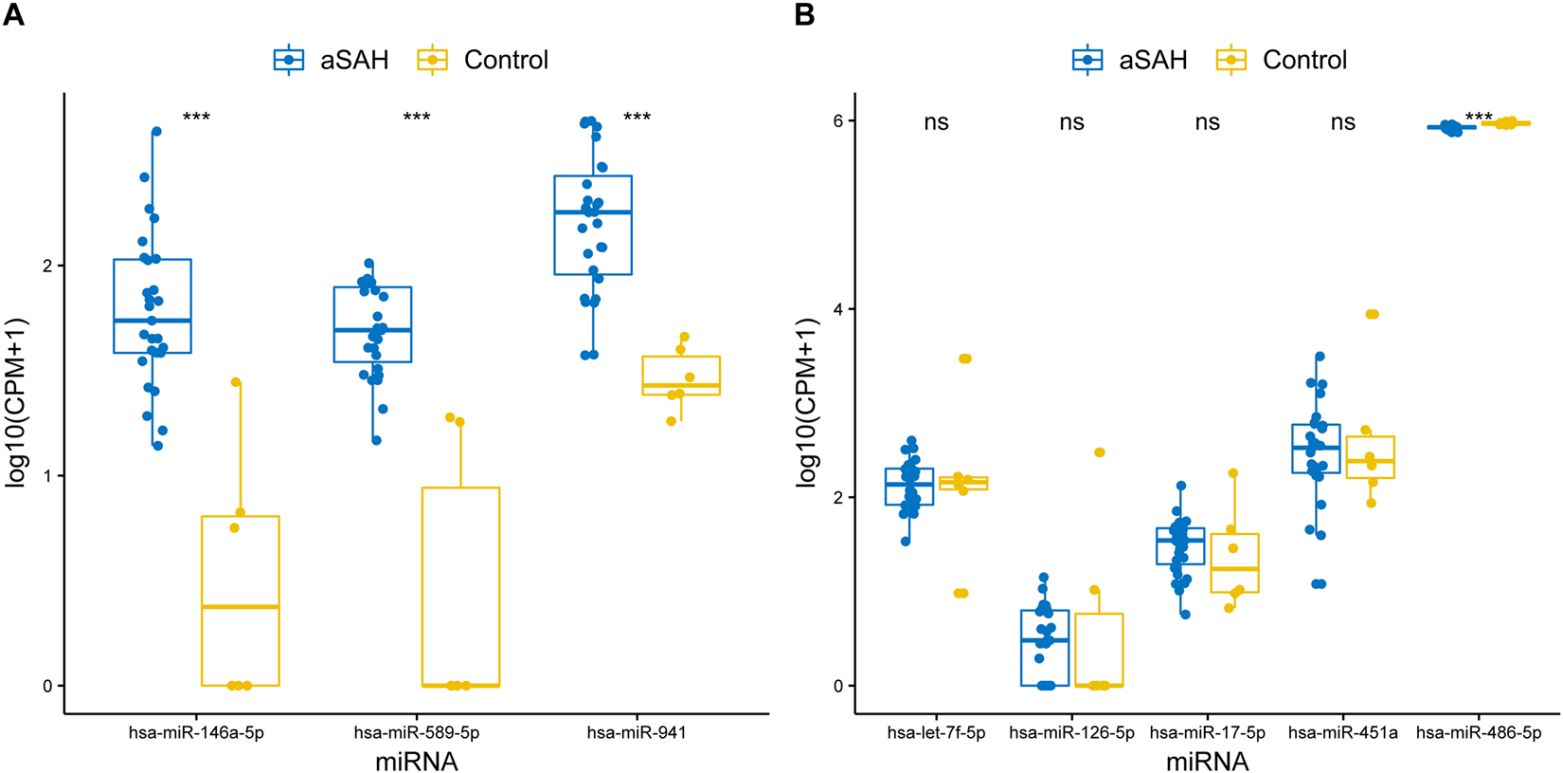
Expression levels of DE miRNAs between groups. The x-axis shows the miRNA and y-axis represent the expression in log10 scale. Data is normalized as counts per million (CPM). Blue colour represents aSAH patients and yellow represents control group. Three asterisks (***) indicate if the data is statistically significant (p-value < 0.001) according to Wilcoxon test; ns is not significant. (**A**) Up-regulated miRNAs. (**B**) Down-regulated miRNAs.

The miRNAs *hsa-miR-589-5p* and *hsa-miR-941* are still poorly described and found in few related studies. The *hsa-miR-589-5p* is described in a list of miRNAs induced by hypoxia, that contributes to the pathogenesis of various human diseases including hypertension, stroke and myocardial or cerebral infarction^62^. Additionally, the *hsa-miR-589-5p* was described in “The colorectal microRNAome”^63^ and in a “mammalian microRNA expression atlas”^64^. Regarding the miRNA *hsa-miR-941*, it has been described in cervical cancer^65^, human embryonic stem cells and neural precursors^66^, and was described as remained significantly associated with incident stroke risk in a study of “Stroke and Circulating Extracellular RNAs”^67^. More detailed analysis of expression levels between DE miRNAs is shown in Fig. 3. All up-regulated miRNA (*hsa-miR-146a-5p, hsa-miR-589-5p* and *hsa-miR-941)* showed a significant difference (p-value < 0.001 using Wilcoxon test) between expression levels of patient and control groups (Fig. 3A). Conversely, for the five down-regulated miRNAs *let-7f-5p, hsa-miR-126-5p, hsa-miR-17-5p, hsa-miR-451a*, and *hsa-miR-486-5p*, significant difference was found only for *hsa-miR-486-5p* (Fig. 3B).

### Statistical power analysis

Several technical and biological aspects have effect on the variability of RNA-seq experiments^68^. For an inferential analysis, depending on the desired statistical power, a minimum of three biological replicates is usually enough to achieve reliable results^69^. For a differential expression analysis, it has been suggested that increasing the number of replicates is preferable over increasing the sequencing depth^70,71^, with studies recommending from six to 12 biological replicates^72^. Regarding specifically circulating miRNA studies for biomarkers discovery, it has been shown that small sample sizes can lead to an increase in false positive and false negative results, with studies suggesting at least 7 samples in order to detect with at least 80% of power a 2-fold change in expression levels with a false discovery rate (FDR) of 10%^73^.

Here we analysed sets of unpaired sample groups with a significant difference in number of replicates. Despite having a considerable number of aSAH case samples (n = 27), our control group size was restricted (n = 6). Therefore, we measured the statistical power estimated to our data, given parameters such as sequencing depth and sample variation. Power estimation was evaluated using the R package RNASeqPower, that accounts for RNA-seq specificities (e.g. technical variation between different runs) by considering negative binomial distributions^41^. We examined the overall sequencing depths and the coefficient of variation in expression across samples for all eight DE miRNAs found previously. As result we obtained a statistical power of 84% under criteria of at least 2-fold and FDR alpha equal 0.05 (Supplementary Table S1).

### *In silico* identification of miRNA target genes

Target genes for each DE miRNA were retrieved from miRTarBase and DIANA Tools. These two databases accumulate data from different species, and all data are experimentally curated. Database validations are reported by western blot, microarray, and next-generation sequencing experiments. For the eight DE miRNA identified (*let-7f-5p, hsa-miR-126-5p, hsa-miR-146a-5p, hsa-miR-17-5p, hsa-miR-451a, hsa-miR-486-5p, hsa-miR-589-5p* and *hsa-miR-941*), miRTarBase indicates 1764 distinct target genes (Supplementary Material - Additional File 4) and DIANA Tools indicates 819 (Supplementary Material - Additional File 5), with 286 targets in common between both approaches. From those 286, 148 targets were found having at least two miRNAs associated considering the sum of the two databases. Some of these genes include proto-oncogenes (*MYC, MDM2*, and *CRK*); genes related with caspase activation and apoptosis (*PMAIP1*, *BCL2*); cyclin kinase regulators (*CCND1*, *CDKN1A*), and growth factors (*TGFBR2*, *VEGFA*). Interestingly, two target genes (*THBS1* and *VEGFA*) are related with aSAH, due to their association with thrombospondin and vascular endothelial growth factor (Table 3).

**Table 3:** Main DE miRNA target genes identified using miRTarBase and DIANA Tools.

Gene name^α^	miRNAs	Gene description
*MDM2*	*hsa-miR-17-5p* *hsa-let-7f-5p* *hsa-miR-126-5p* *hsa-miR-146a-5p* *hsa-miR-589-5p*	*MDM2* proto-oncogene
*PMAIP1*	*hsa-miR-146a-5p* *hsa-miR-589-5p* *hsa-let-7f-5p* *hsa-miR-126-5p* *hsa-miR-17-5p*	phorbol-12-myristate-13-acetate-induced protein 1
*CRK*	*hsa-miR-17-5p* *hsa-let-7f-5p* *hsa-miR-126-5p* *hsa-miR-589-5p*	*CRK* proto-oncogene, adaptor protein
***MYC***	*hsa-miR-17-5p* *hsa-miR-451a* *hsa-let-7f-5p* *hsa-miR-126-5p*	*MYC* proto-oncogene, *bHLH* transcription factor
***BCL2***	*hsa-miR-17-5p* *hsa-miR-451a* *hsa-miR-126-5p*	*BCL2*, apoptosis regulator
***CCND1***	*hsa-miR-17-5p* *hsa-let-7f-5p* *hsa-miR-126-5p*	cyclin D1
***CDKN1A***	*hsa-miR-17-5p* *hsa-miR-146a-5p* *hsa-let-7f-5p*	cyclin dependent kinase inhibitor 1A
*PIK3CA*	*hsa-miR-17-5p* *hsa-miR-126-5p* *hsa-miR-451a*	phosphatidylinositol-4,5-bisphosphate 3-kinase catalytic subunit alpha
***PTGS2***	*hsa-miR-146a-5p* *hsa-miR-589-5p* *hsa-let-7f-5p*	prostaglandin-endoperoxide synthase 2
*RAC1*	*hsa-miR-146a-5p* *hsa-let-7f-5p* *hsa-miR-17-5p*	ras-related C3 botulinum toxin substrate 1 (rho family, small GTP binding protein Rac1)
*RBL1*	*hsa-miR-17-5p* *hsa-miR-146a-5p* *hsa-miR-589-5p*	RB transcriptional corepressor like 1
*TGFBR2*	*hsa-miR-17-5p* *hsa-let-7f-5p* *hsa-miR-126-5p*	transforming growth factor beta receptor 2
*VEGFA*	*hsa-miR-17-5p* *hsa-miR-126-5p* *hsa-miR-146a-5p*	vascular endothelial growth factor A
*THBS1*	*hsa-miR-17-5p* *hsa-let-7f-5p*	thrombospondin 1

^α^Also found with mirTargetLink (strong evidence target).

DIANA Tools also provides for each target gene, the association with KEGG Pathway database. Some identified pathways include terms associated with different types of cancer as colorectal, pancreatic and renal; pathways of malaria, fatty acid degradation, PI3K-Akt signalling, focal adhesion, viral carcinogenesis, regulation of actin cytoskeleton, hepatitis B, endocytosis, hippo, *WNT* and sphingolipid signalling, among others (Supplementary Material - Additional File 5).

**Figure 4:**
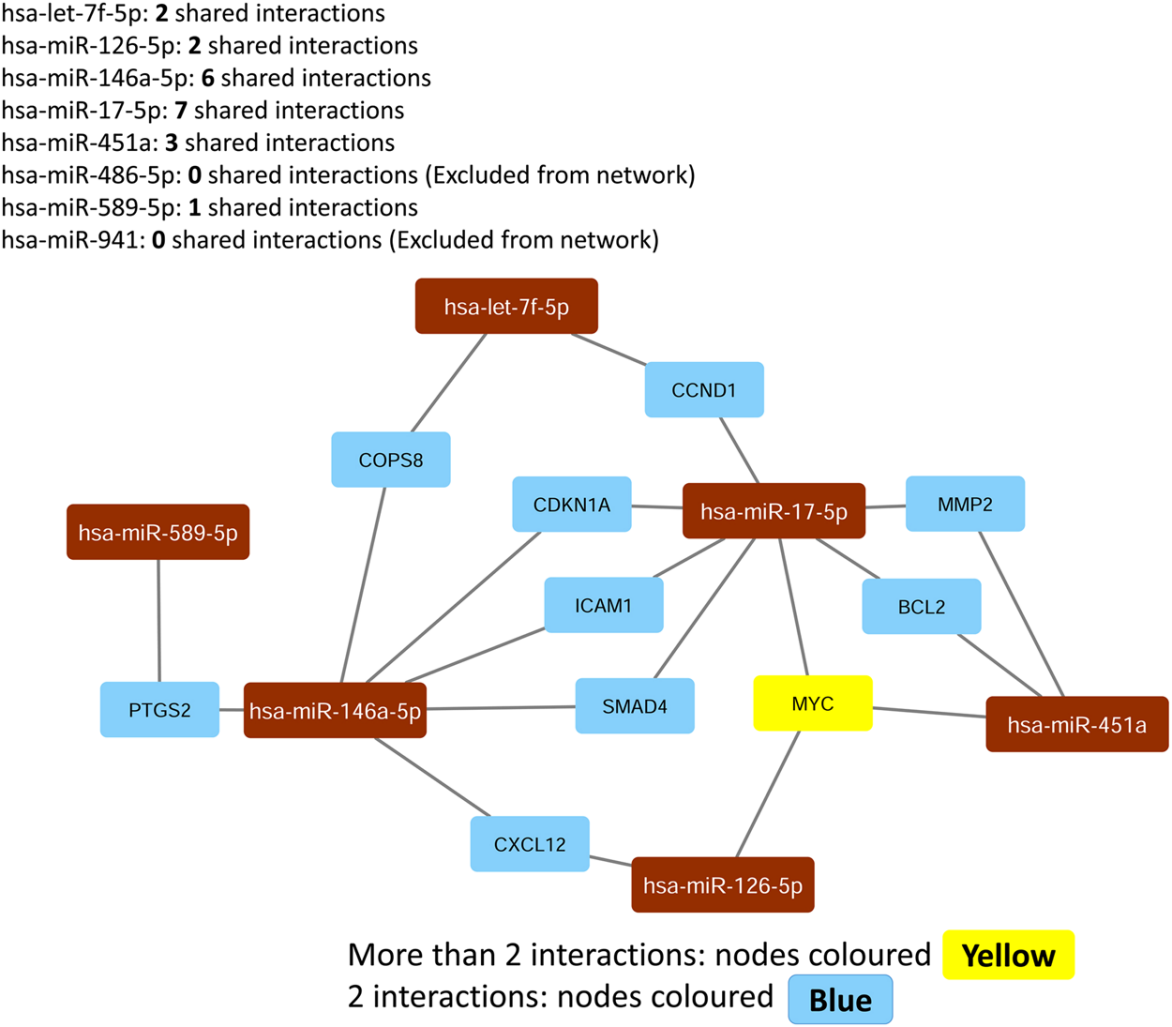
Network of DE miRNAs and targets associated. The figure shows only strong evidence associations indicated by the software mirTargetLink and plotted using Cytoscape. Blue colour represents genes with two miRNA interactions; yellow represents more than two miRNA interactions. Brow rectangles represent the miRNAs.

Associations between miRNA and its target genes were further explored using the mirTargetLink software. Considering only strong evidence targets, ten genes were found targeted by at least two miRNAs (Fig. 4). Among these genes, five were reported as representative by miRTarBase and DIANA Tools (Table 3, in bold). Notably, the *MYC* gene (orange rectangle in Fig. 4), had three down-regulated miRNAs associated: *hsa-miR-451a, hsa-miR-17-5p*, and *hsa-miR-126-5p*. This gene was also reported by DIANA and miRTarBase (Table 3), suggesting involvement with aneurysmatic subarachnoid haemorrhage*. MYC* is known for being involved in cell growth, apoptosis, and metabolism^74^, and was also described as an oncogenic transcription factor^75^. High-level expression of *MYC* can favour cell proliferation, inhibit cell differentiation and contributes to tumorigenesis^76,77^.

### Novel miRNA prediction

**Figure 5:**
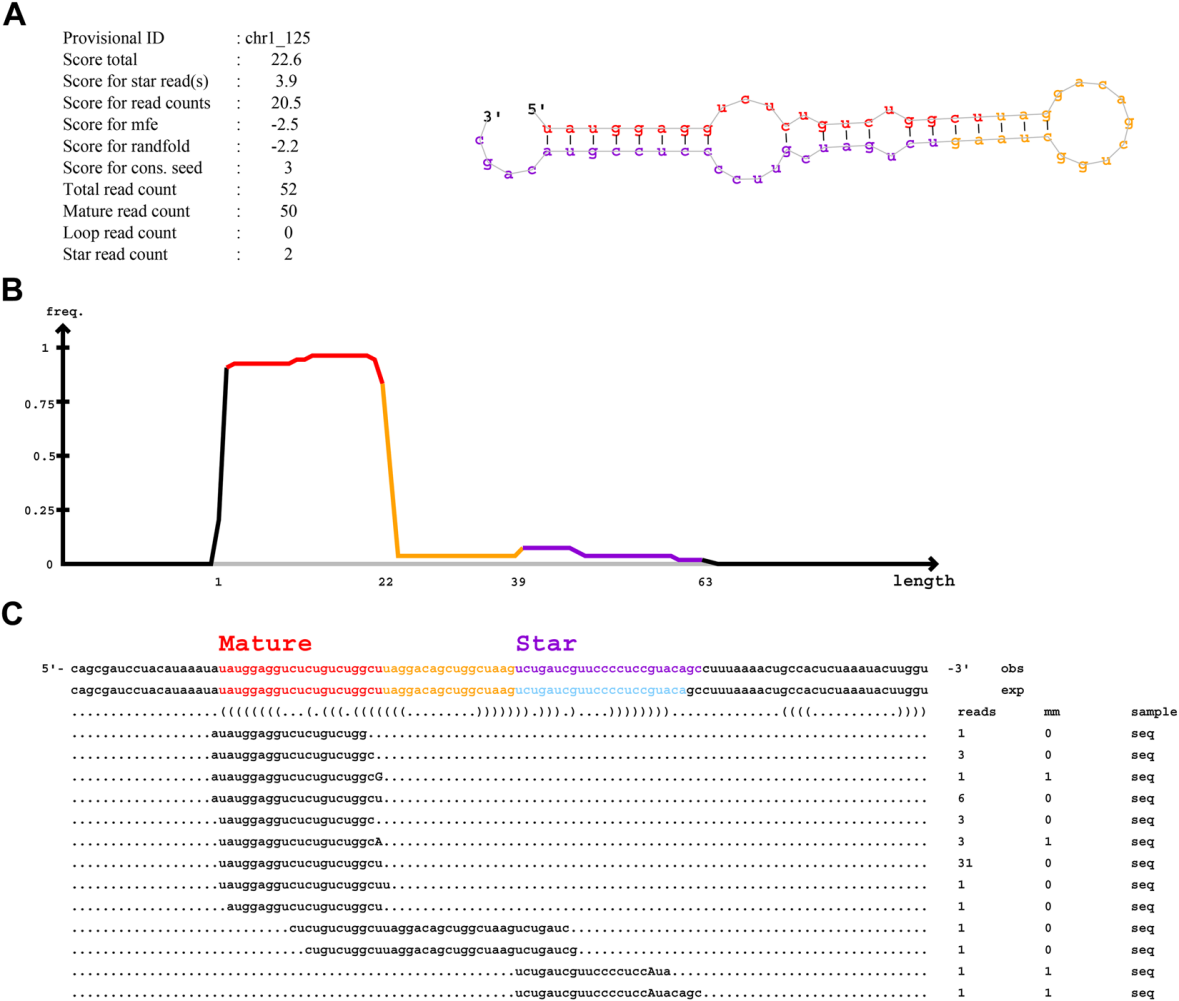
Possible novel miRNA identified by mirDeep2. This molecule is found only in 11 samples from aSAH patients, not present in the control samples. (**A**) Information and possible secondary structure of this novel miRNA. (**B**) Frequency of reads aligned in each pre-miRNA region. (**C**) Read alignment.

The use of Next Generation Sequencing (NGS) technologies provides an excellent approach to identify possible novel molecules. *In silico* prediction of novel miRNAs was performed using the software mirDeep2. We found 33 potential novel miRNAs with a score higher than 5 (Supplementary Material - Additional File 6). Regions mapped with small differences in the beginning or end of alignments (1–4 nucleotides) were merged and considered being the same miRNA. As a result, 15 miRNAs were identified in distinct positions of the human genome with evidence of reads in 15 samples (Supplementary Material - Additional File 7). These possible novel miRNAs were found only in the aSAH samples, and not in control participants. The number of reads mapped in these molecules varies up to 52 depending on samples (Supplementary Fig. S5). The predicted pre-miRNA with the highest number (52) of mapped reads in a sample is shown in Fig. 5. This miRNA (provisional ID “ch1_125”) is located on chromosome 1 and present evidence of expression in a total of 11 samples from aSAH patients (Supplementary Fig. S5). The predicted secondary structures of the other predicted molecules are available in Supplementary Material - Additional File 8.

## Conclusions

A global miRNA expression analysis profile of patients after aneurysmal subarachnoid haemorrhage (aSAH) was conducted using small RNA deep-sequencing on an Illumina MiSeq platform. Eight miRNAs were found differentially expressed in aSAH patients, three being up-regulated: *hsa-miR-146a-5p, hsa-miR-589-5p, and hsa-miR-941;* and five being down-regulated*: let-7f-5p, hsa-miR-126-5p, hsa-miR-17-5p, hsa-miR-451a*, and *hsa-miR-486-5p*. Moreover, 148 target genes were identified associated with at least two DE miRNAs indicated by two different approaches. The *MYC* gene was found having the highest number of strong-related DE miRNAs associated to its regulation, suggesting an important target involved in a complex of subarachnoid haemorrhage. Additionally, 15 potential novel molecules of miRNAs were found occurring only in samples from aSAH patients, as novel players in this complex disease. One specifically, mapped to the human chromosome 1, present in 11 aSAH samples showed an elevated number of reads mapped. These results provide resources for future researchers for identifying novel biomarkers to help the clinical management of patients with a predisposition to develop an aneurysmal subarachnoid haemorrhage.

## Electronic supplementary material

Supplementary material

Additional File 1

Additional File 2

Additional File 3

Additional File 4

Additional File 5

Additional File 6

Additional File 7

Additional File 8
