# Supplementary material for: Global miRNA expression profile reveals novel molecular players in aneurysmal subarachnoid haemorrhage
Source: Sci Rep. 2018 Jun 8;8:8786. doi: 10.1038/s41598-018-27078-w (PMC5993784; doi:10.1038/s41598-018-27078-w)
Supplement: Supplementary file 1 — Supplementary material [file 41598_2018_27078_MOESM1_ESM.pdf]

Supplementary Material

Global miRNA expression profile reveals novel molecular players in aneurysmal subarachnoid haemorrhage

Katia de Paiva Lopes[1,†], Tatiana Vinasco-Sandoval[1,2,†], Ricardo Assunção Vialle[1,†], Fernando Mendes Paschoal Junior[3], Vanessa Albuquerque P. Aviz Bastos[4], Edson Bor-Seng-Shu[5], Manoel Jacobsen Teixeira[5], Elizabeth Sumi Yamada[2,6,7], Pablo Pinto[2], Amanda Ferreira Vidal[1,2], Arthur Ribeiro-dos-Santos[1], Fabiano Moreira[1,2], Sidney Santos[1,2], Eric Homero Albuquerque Paschoal[4,7], Ândrea Ribeiro-dos-Santos*[,1,2,7]

1 Laboratório de Genética Humana e Médica, Programa de Pós-Graduação em Genética e Biologia Molecular, Universidade Federal do Pará, Belém, Brazil.
2 Núcleo de Pesquisas em Oncologia, Programa de Pós-Graduação em Oncologia e Ciências Médicas, Universidade Federal do Pará, Belém, Brazil.
3 Serviço de Neurocirurgia - Hospital Ophir Loyola, Unidade Neuromuscular do Complexo Hospitalar da UFPA, Belém, Brazil.
4 Serviço de Neurofisiologia Intraoperatória, Neurogenesis Instituto de Neurociências, Belém, Brazil.
5 Serviço de Neurocirurgia do Hospital das Clínicas da Faculdade de Medicina da USP, São Paulo, Brazil.
6 Laboratório de Neuropatologia Experimental, Universidade Federal do Pará, Belém, Brazil.
7 Grupo de Pesquisa Amazônia Neurovascular, Universidade Federal do Pará, Belém, Brazil.

† Authors contributed equally to this work.

* Corresponding author: Ândrea Ribeiro-dos-Santos (akelyufpa@gmail.com).

This PDF file includes 5 supplementary figures and 2 supplementary table:

Supplementary Figure S1: Correlation of miRNA expression by sample.

Supplementary Figure S2: Principal component analysis comprising aSAH patients and control individuals.

Supplementary Figure S3: Statistics of DE miRNAs between group1 and control.

Supplementary Figure S4: Statistics of DE miRNAs between group2 and control.

Supplementary Figure S5: Total number of reads mapped to novel miRNAs by sample.

Supplementary Table S1: Statistical power analysis for sample sizes.

Supplementary Table S2: DE miRNAs between aSAH patients and control group from edgeR software.

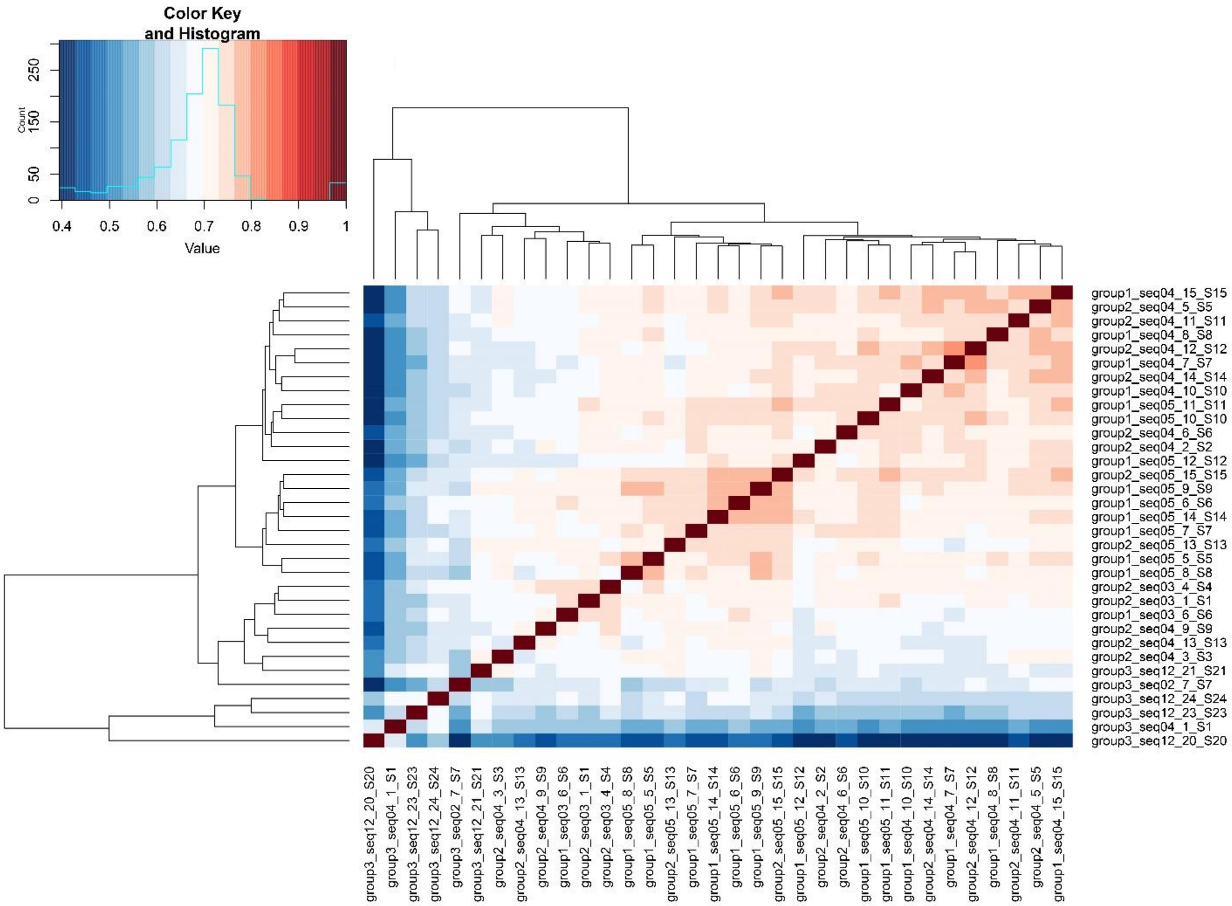

Supplementary Figure S1: Correlation of miRNA expression by sample. Heatmap shows Spearman correlation of miRNA expression of 33 samples comprising aSAH patients in group1 (n = 14), group2 (n = 13) and control individuals (n = 6). **Dark-red** colors correspond to maximum correlation, **dark-blue** colors minimum correlation and white corresponds to average correlation.

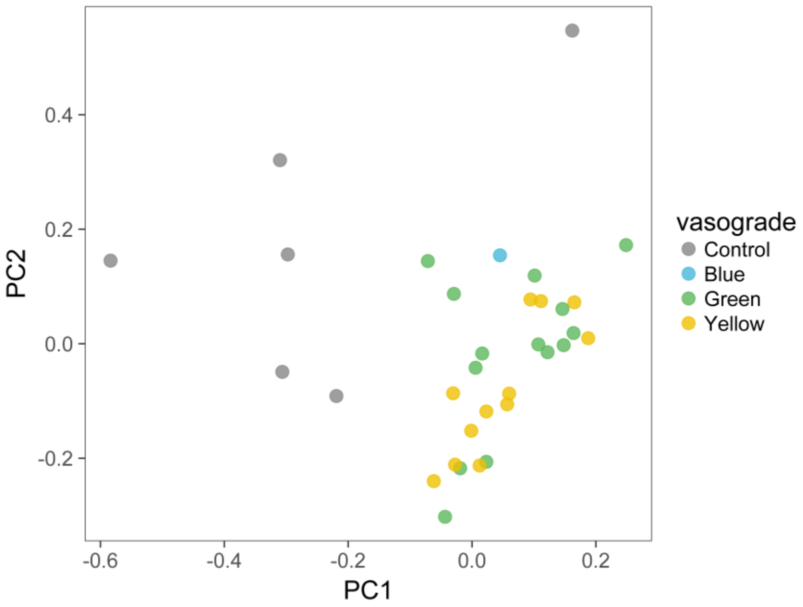

Supplementary Figure S2: Principal component analysis comprising aSAH patients and control individuals. The dots are colored according to vasograde scale described in Table 1 of clinical parameters (main article).

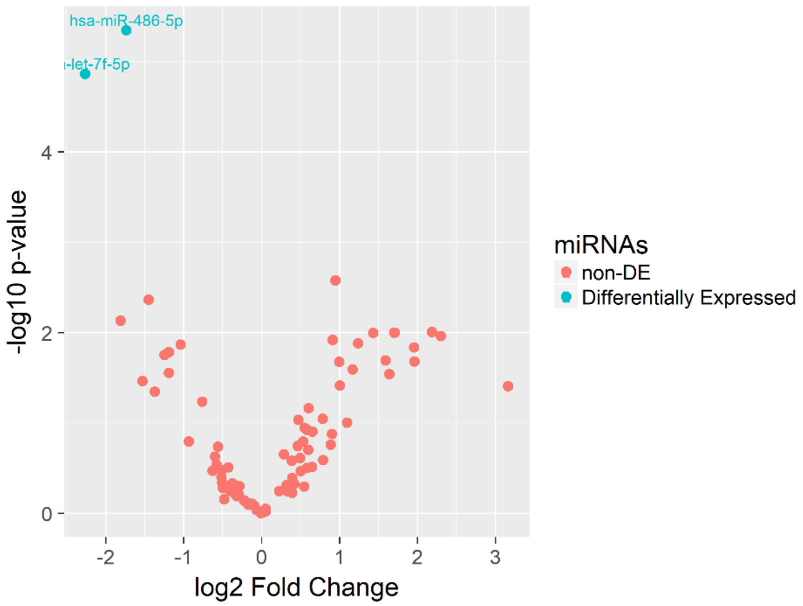

Supplementary Figure S3: Statistics of DE miRNAs between group1 and control. The x-axis represents the values of log2(fold change) and y-axis are p-value in the scale of log10. Blue dots are the DE miRNAs, and red dots are non-DE miRNAs.

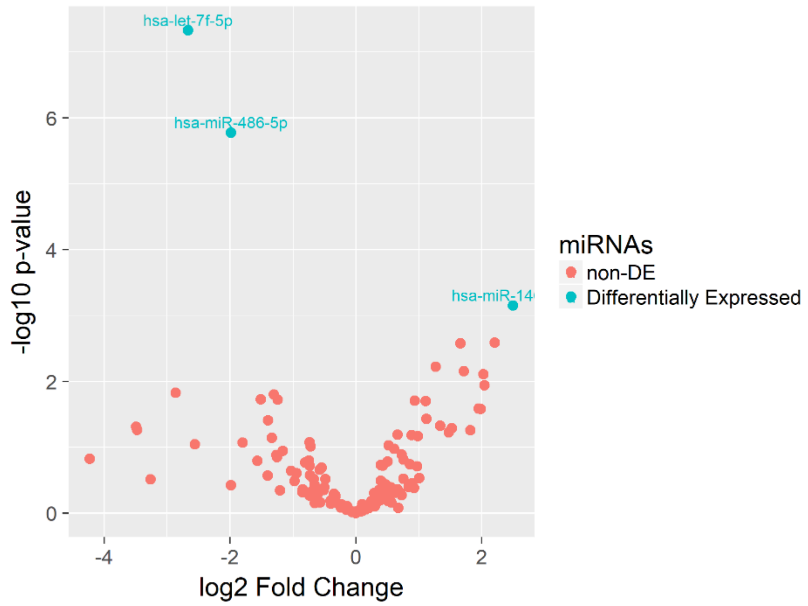

Supplementary Figure S4: Statistics of DE miRNAs between group2 and control. The x-axis represents the values of log2(fold change) and y-axis are p-value in the scale of log10. Blue dots are the DE miRNAs, and red dots are non-DE miRNAs.

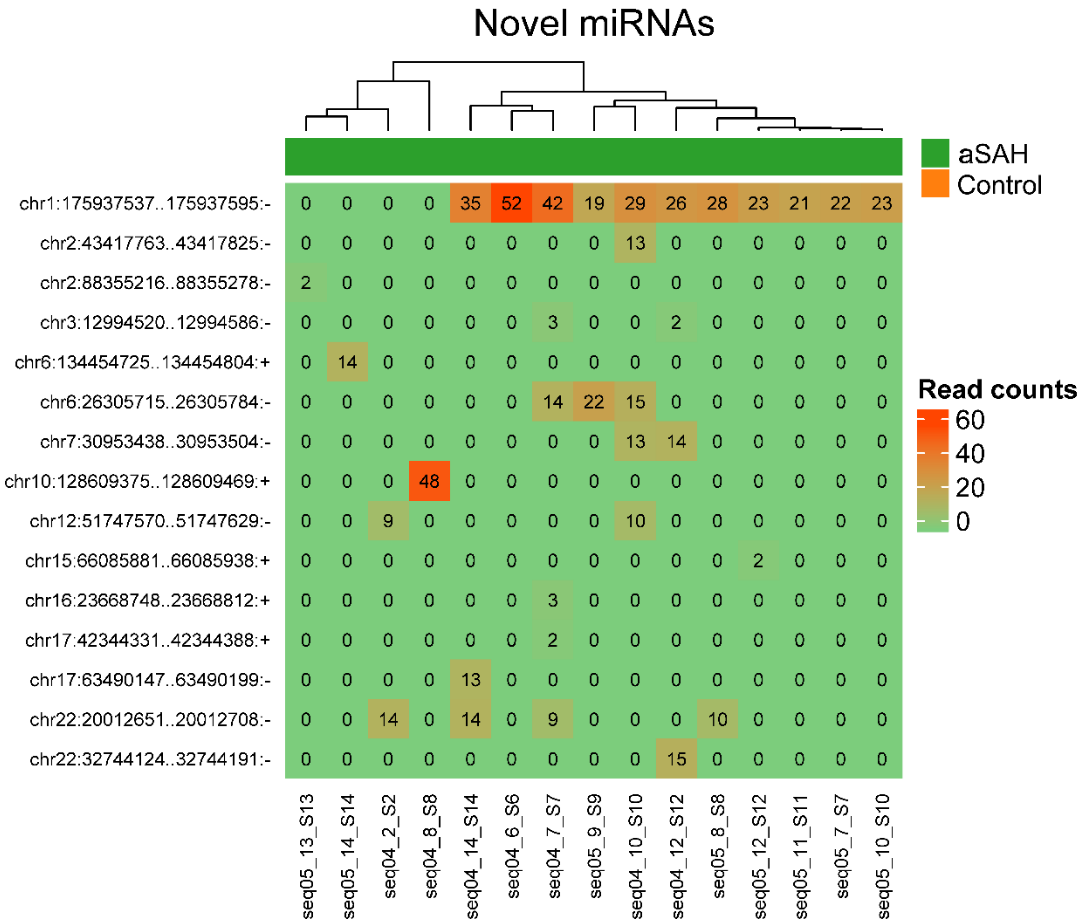

Supplementary Figure S5: Total number of reads mapped to novel miRNAs by sample. Novel miRNAs were identified using miRDeep2 with a score higher than 5. miRNAs with overlapping regions were merged, resulting in 15 final miRNAs (showed in Figure). Top bar represents sample group (aSAH patients and control individuals). X-axis: sample name; Y-axis: chromosome position. Heatmap **green** color indicates 0 or few reads; **Orange**: Maximum number of reads aligned in that position.

Supplementary Table S1: Statistical power analysis for sample sizes

Fold change	Power		
	FDR α<0.1	FDR α<0.05	FDR α<0.01
**1.25**	0.2440176	0.1565958	0.05214386
**1.5**	0.5334426	0.4085860	0.19848390
**1.75**	0.7707074	0.6649709	0.42473400
**2**	0.9049893	0.8402304	0.64785659

Note: power values were estimated using RNASeqPower R package considering unpaired sample sizes of 27 aSAH patients and 6 controls and the average expression data of eight differentially expressed miRNAs found using DESeq2 (see Methods section for details).

Supplementary Table S2: DE miRNAs between aSAH patients and control individuals identified with edgeR software

miRNA[α]	log2FoldChange	log(CPM)[β]	Adjusted *p*-value	*p*-value
hsa-miR-15b-3p	6.918817	3.503655	1.11E-15	1.11E-15
hsa-miR-142-5p	6.621679	7.045975	7.05E-22	7.05E-22
hsa-miR-1306-5p	-5.00408	3.175254	0.002968	0.002968
hsa-miR-3200-3p	-5.00224	3.188815	0.004988	0.004988
hsa-miR-503-5p	-4.50662	2.841098	0.031649	0.031649
hsa-miR-374b-5p	4.477083	2.004457	5.72E-08	5.72E-08
hsa-miR-126-5p	**4.291935**	**3.640621**	**4.01E-13**	**4.01E-13**
hsa-miR-122-5p	3.946439	2.115645	4.19E-07	4.19E-07
hsa-miR-16-2-3p	3.801885	2.243513	0.00031	0.00031
hsa-miR-144-5p	3.54505	4.863662	7.60E-12	7.60E-12
hsa-miR-769-5p	-3.53025	4.262203	0.000853	0.000853
hsa-miR-664a-3p	2.999453	2.371334	2.62E-05	2.62E-05
hsa-miR-185-3p	-2.82041	4.479448	0.000997	0.000997
hsa-miR-942-5p	-2.68145	4.933603	0.000502	0.000502
hsa-miR-146a-5p	**-2.41456**	**5.845715**	**0.000186**	**0.000186**
hsa-miR-16-5p	2.382268	9.082687	1.28E-08	1.28E-08
hsa-miR-93-3p	2.362859	2.748328	0.000512	0.000512
hsa-miR-500a-3p	-2.34557	5.025987	0.000498	0.000498
hsa-miR-19b-3p	2.343083	3.363962	8.54E-05	8.54E-05
hsa-miR-222-3p	-2.30259	4.982155	0.000383	0.000383
hsa-miR-100-5p	2.098872	3.463587	0.027679	0.027679
hsa-let-7f-5p	**2.0946**	**7.682596**	**4.75E-09**	**4.75E-09**
hsa-miR-589-5p	**-2.05657**	**5.468522**	**0.000448**	**0.000448**
hsa-miR-425-5p	2.010978	7.201045	1.64E-06	1.64E-06
hsa-miR-324-5p	-1.86763	4.626887	0.017297	0.017297
hsa-miR-486-5p	**1.864753**	**20.10242**	**2.44E-10**	**2.44E-10**
hsa-miR-196b-5p	-1.83641	3.470993	0.044455	0.044455
hsa-miR-451a	**1.826463**	**9.238277**	**0.000465**	**0.000465**
hsa-miR-130b-5p	-1.73826	3.876097	0.040365	0.040365
hsa-miR-941	**-1.70564**	**7.29286**	**0.003041**	**0.003041**
hsa-miR-5010-3p	1.574023	2.593417	0.038678	0.038678
hsa-miR-185-5p	1.559669	6.777627	2.20E-05	2.20E-05
hsa-miR-3158-3p	-1.53803	5.69166	0.0175	0.0175
hsa-miR-148a-3p	-1.49503	5.478709	0.02025	0.02025
hsa-miR-186-5p	-1.47238	8.682725	0.002121	0.002121
hsa-miR-126-3p	1.455186	3.167767	0.038516	0.038516
hsa-miR-423-3p	-1.3496	8.817697	0.0058	0.0058
hsa-miR-221-3p	-1.30789	6.821661	0.003564	0.003564
hsa-miR-324-3p	-1.24013	4.561812	0.048358	0.048358
hsa-miR-10a-5p	1.235061	3.965005	0.010349	0.010349
hsa-miR-27b-3p	-1.1963	6.35751	0.004691	0.004691
hsa-miR-15a-5p	1.185337	4.099445	0.008867	0.008867
hsa-miR-182-5p	-1.14964	11.30623	0.000711	0.000711
hsa-miR-574-3p	1.144766	5.511526	0.04399	0.04399
hsa-miR-17-5p	**1.088197**	**5.144431**	**0.007741**	**0.007741**
hsa-miR-151a-3p	1.030884	6.28436	0.010824	0.010824
hsa-miR-128-3p	-1.0236	5.31245	0.035364	0.035364

[α] miRNAs also found by DESeq2 are indicated in bold.
[β] log(CPM) represents log2 of counts-per-million (see Methods section).
