# Supplementary material for: Global miRNA expression profile reveals novel molecular players in aneurysmal subarachnoid haemorrhage
Source: Sci Rep. 2018 Jun 8;8:8786. doi: 10.1038/s41598-018-27078-w (PMC5993784; doi:10.1038/s41598-018-27078-w)
Supplement: Supplementary file 9 — Additional File 8 [file 41598_2018_27078_MOESM9_ESM.zip › Additional_File-8.pdf]

Provisional ID	:	chr12_1171
Score total	:	8.8
Score for star read(s)	:	3.9
Score for read counts	:	-1.3
Score for mfe	:	1.7
Score for randfold	:	1.6
Score for cons. seed	:	3
Total read count	:	9
Mature read count	:	8
Loop read count	:	0
Star read count	:	1

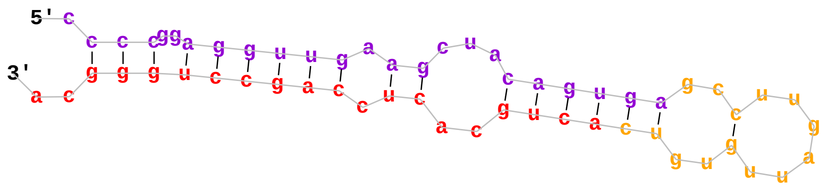

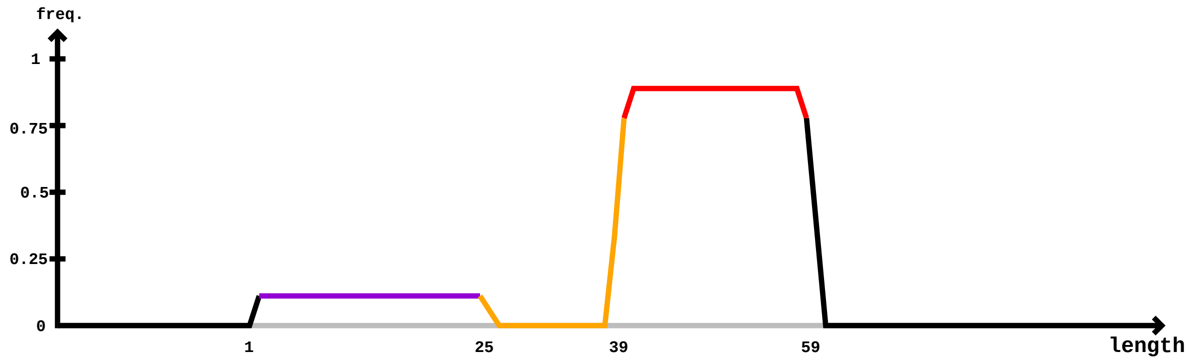


```
                    Star                            Mature
5'- ggugggaggauugcuuguuccccggagguugaagcuacagugagccuugauugugucacugcacuccagccugggcaacagguaagacucugucucaaaaaaaaacaaaaaag   -3'   obs
    ggugggaggauugcuguucccggagguugaagcuacagugagccuugauugugucacugcacuccagccugggcaacagguaagacucugucucaaaaaaaaacaaaaaag         exp
    ((..((((..(((((((((.(((..((((((.((...((((((..(......)..))))))..)).))))))))).)))))))))..))))..))..................   reads    mm    sample
    ...................ccccggagguugaagcuacagugU......................................................................   1        1     seq
    ........................................................cacugcacuccagccugggc.....................................   1        0     seq
    ........................................................cacugcacuccaAccugggca....................................   1        1     seq
    ........................................................cacugcacuccagccugggcGa...................................   1        1     seq
    .........................................................acugcacuccagccugggca....................................   2        0     seq
    .........................................................acugcacuccagccugggcGa...................................   2        1     seq
    ..........................................................cugcacuccagccugggcGa...................................   1        1     seq
```

Provisional ID	:	chr22_1949
Score total	:	8.6
Score for star read(s)	:	3.9
Score for read counts	:	1.1
Score for mfe	:	2.5
Score for randfold	:	1.6
Score for cons. seed	:	-0.6
Total read count	:	14
Mature read count	:	7
Loop read count	:	0
Star read count	:	7

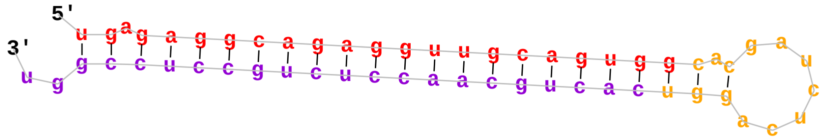

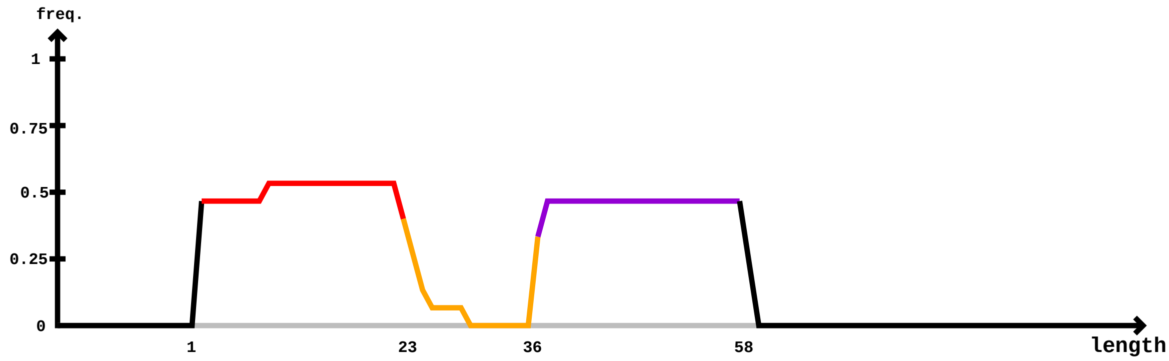

Mature **Star**

```
5'- aucgcauugaaccugagaggcagagguugcaguggcacgaucucaggucacugcaaccucugccuccgguauucaagcgauucuccugccucagccuuuugaggugacaaa  -3'  obs
    aucgcauugaaccugagaggcagagguugcaguggcacgaucucaggucacugcaaccucugccuccgguauucaagcgauucuccugccucagccuuuugaggugacaaa       exp
    (((((.(((((.(((.((((((((((((((((((((.(.......))))))))))))))))))))))))..))))))))))......(((((((....)))))))......  reads  mm  sample
    .............ugagaggcagagguugcagug.............................................................................  2  0  seq
    .............ugagaggcagagguugcagugg............................................................................  3  0  seq
    .............ugagaggcagagguugcaguggc...........................................................................  1  0  seq
    .............ugagaggcagagguugcaguggAa..........................................................................  1  1  seq
    ....................cagagguugcaguggcacgau......................................................................  1  0  seq
    ................................................cacugcaaccucugccuccggu.........................................  2  0  seq
    ................................................cacugcaaccucugccuccggA.........................................  1  1  seq
    ................................................cacugcaaccucugccuccggAa........................................  2  1  seq
    .................................................acugcaaccucugccuccggu.........................................  2  0  seq
```

Provisional ID	:	chr1_125
Score total	:	22.6
Score for star read(s)	:	3.9
Score for read counts	:	20.5
Score for mfe	:	-2.5
Score for randfold	:	-2.2
Score for cons. seed	:	3
Total read count	:	52
Mature read count	:	50
Loop read count	:	0
Star read count	:	2

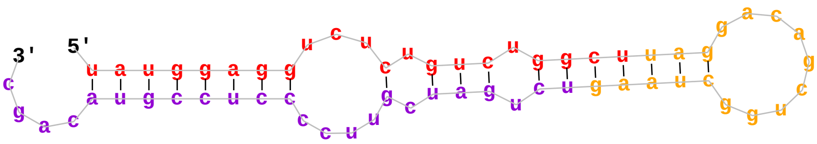

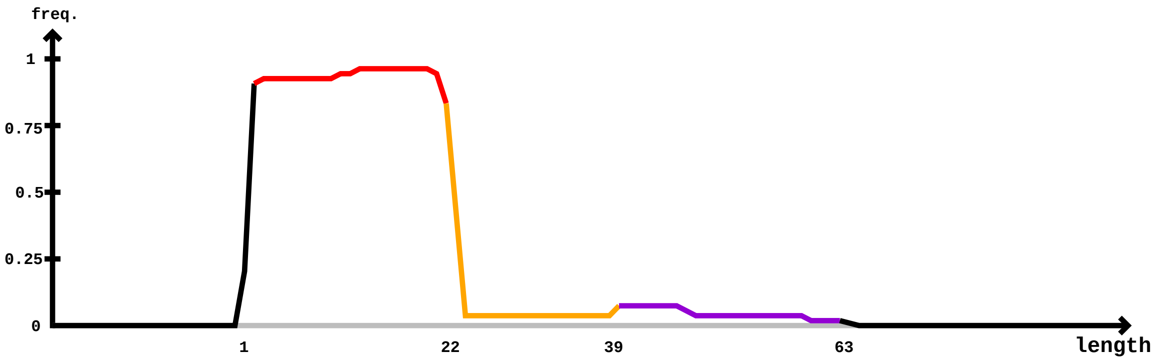

Mature **Star**

```
5'- cagcgauccuacauaaauauauggaggucucugucuggcuuaggacagcuggcuaagucugaucguuccccuccguacagccuuuaaaacugccacucuaaauacuuggu   -3'   obs
    cagcgauccuacauaaauauauggaggucucugucuggcuuaggacagcuggcuaagucugaucguuccccuccguacagccuuuaaaacugccacucuaaauacuuggu         exp
    ...................((((((((...(.(((.(((((((.........))))))).))).)....))))))))..............((((...........))))   reads   mm   sample
    ..................auauggaggucucugucugg........................................................................   1       0    seq
    ..................auauggaggucucugucuggc.......................................................................   3       0    seq
    ..................auauggaggucucugucuggcG......................................................................   1       1    seq
    ..................auauggaggucucugucuggcu......................................................................   6       0    seq
    ...................uauggaggucucugucuggc.......................................................................   3       0    seq
    ...................uauggaggucucugucuggcA......................................................................   3       1    seq
    ...................uauggaggucucugucuggcu......................................................................   31      0    seq
    ...................uauggaggucucugucuggcuu.....................................................................   1       0    seq
    ....................auggaggucucugucuggcu......................................................................   1       0    seq
    ............................cucugucuggcuuaggacagcuggcuaagucugauc..............................................   1       0    seq
    ..............................cugucuggcuuaggacagcuggcuaagucugaucg.............................................   1       0    seq
    .........................................................ucugaucguuccccuccAua.................................   1       1    seq
    .........................................................ucugaucguuccccuccAuacagc.............................   1       1    seq
```

Provisional ID	:	chr1_245
Score total	:	17.5
Score for star read(s)	:	3.9
Score for read counts	:	15.4
Score for mfe	:	-2.5
Score for randfold	:	-2.2
Score for cons. seed	:	3
Total read count	:	42
Mature read count	:	36
Loop read count	:	0
Star read count	:	6

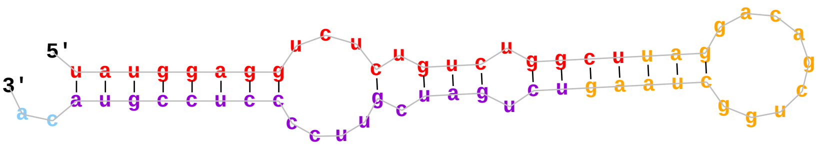

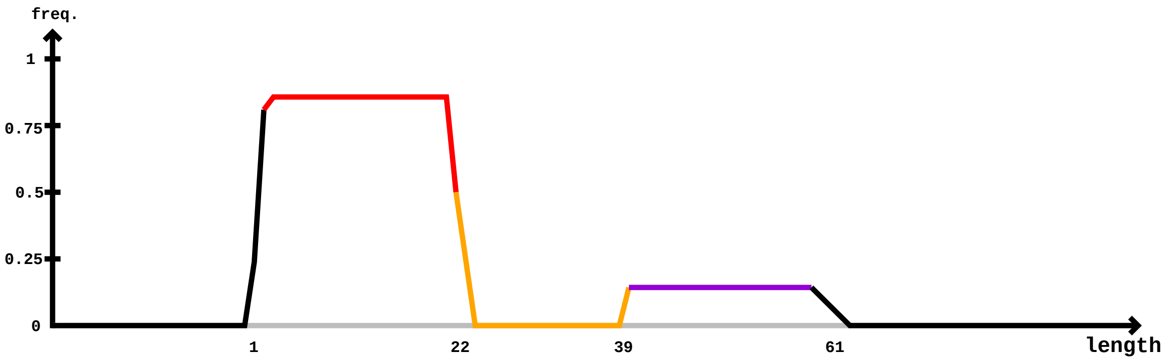

Mature **Star**

```
5'- gcagcgauccuacauaaauauauggaggucucugucuggcuuaggacagcuggcuaagucugaucguuccccuccguacagccuuuaaaacugccacucuaaauacuuggu   -3'    obs
    gcagcgauccuacauaaauauauggaggucucugucuggcuuaggacagcuggcuaagucugaucguuccccuccguacagccuuuaaaacugccacucuaaauacuuggu          exp
    ....................((((((((...(.(((.(((((((.........))))))).))).)....))))))))..............((((...........))))   reads     mm       sample
    ...................auauggaggucucugucuggc.......................................................................   5         0        seq
    ...................auauggaggucucugucuggcu......................................................................   4         0        seq
    ...................auauggaggucucugucuggcG......................................................................   1         1        seq
    ....................uauggaggucucugucuggc.......................................................................   9         0        seq
    ....................uauggaggucucugucuggA.......................................................................   1         1        seq
    ....................uauggaggucucugucuggcA......................................................................   1         1        seq
    ....................uauggaggucucugucuggcu......................................................................   12        0        seq
    ....................uauggaggucucugucuggcuu.....................................................................   1         0        seq
    .....................auggaggucucugucuggcu......................................................................   1         0        seq
    .....................auggaggucucugucuggcuu.....................................................................   1         0        seq
    ..........................................................ucugaucguuccccuccAua.................................   4         1        seq
    ..........................................................ucugaucguuccccuccAuaca...............................   2         1        seq
```

Provisional ID	:	chr3_552
Score total	:	6.2
Score for star read(s)	:	3.9
Score for read counts	:	-4.4
Score for mfe	:	2.2
Score for randfold	:	1.6
Score for cons. seed	:	3
Total read count	:	3
Mature read count	:	2
Loop read count	:	0
Star read count	:	1

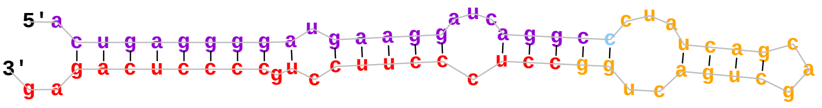

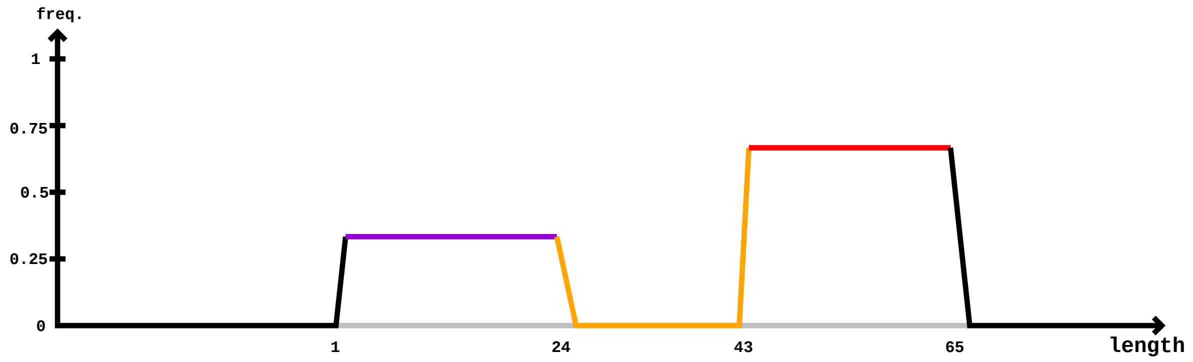


```
                                     Star                                      Mature
5'- gaaacugucccuggcugagccccaccuuacugaggggaugaaggaucaggcccuaucagcagcugacuggccucccuuccugccccucagagaacagugaggccccagaggag   -3'    obs
    gaaacugucccuggcugagccccaccuuacugaggggaugaaggaucaggcccuaucagcagcugacuggccucccuuccugccccucagagaacagugaggccccagaggag          exp
    .......(((((((..(.(((.(((.((.(((((((((.(((((...(((((...((((...))))..))))).))))).).)))))))).))...))).)))))))).))).   reads    mm    sample
    ............................acugaggggaugaaggaucaggc..............................................................   1        0     seq
    ......................................................................ccucccuuccugccccucagag.....................   1        0     seq
    ......................................................................ccucccuuccugccccucagaga....................   1        0     seq
```

Provisional ID	:	chr6_898
Score total	:	5.2
Score for star read(s)	:	-1.3
Score for read counts	:	0
Score for mfe	:	1.9
Score for randfold	:	1.6
Score for cons. seed	:	3
Total read count	:	14
Mature read count	:	14
Loop read count	:	0
Star read count	:	0

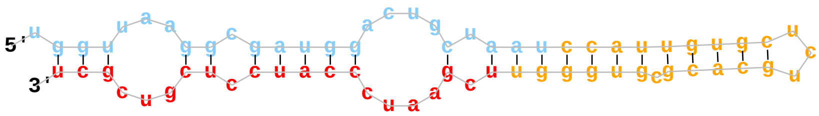

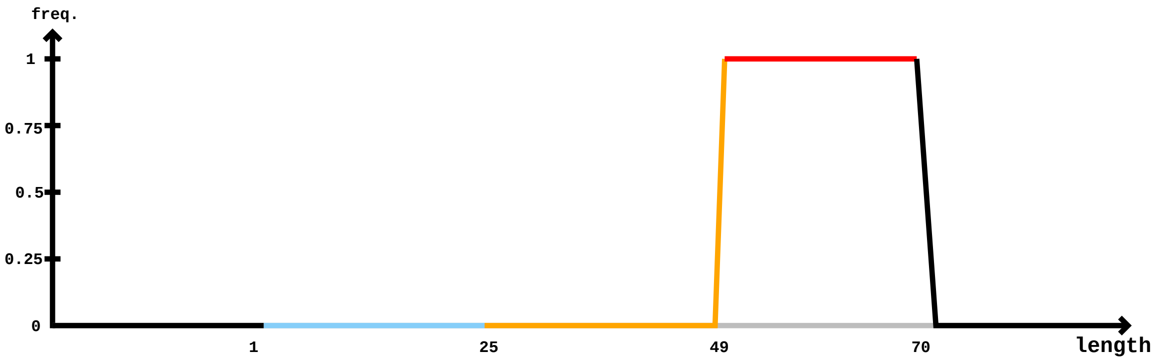

Star Mature

```
5'- ucaggagaggccuggccgagugguuaaggcgauggacugcuaauccauugugcucugcacgcguggguucgaaucccauccucgucgcucggcuuucccugcuaacuggg   -3'    exp
    ((((.(((((...((((((((((....((.(((((....(.((((((((((((...))))).))))))).)....))))))).)))))))))))...))).))..)))).    reads      mm          sample
    ....................................................................ucgaaucccauccucgucgcC.....................    14         1           seq
```

Provisional ID	:	chr16_2033
Score total	:	6.5
Score for star read(s)	:	3.9
Score for read counts	:	-4.4
Score for mfe	:	2.5
Score for randfold	:	1.6
Score for cons. seed	:	3
Total read count	:	3
Mature read count	:	2
Loop read count	:	0
Star read count	:	1

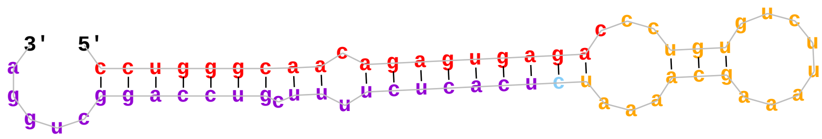

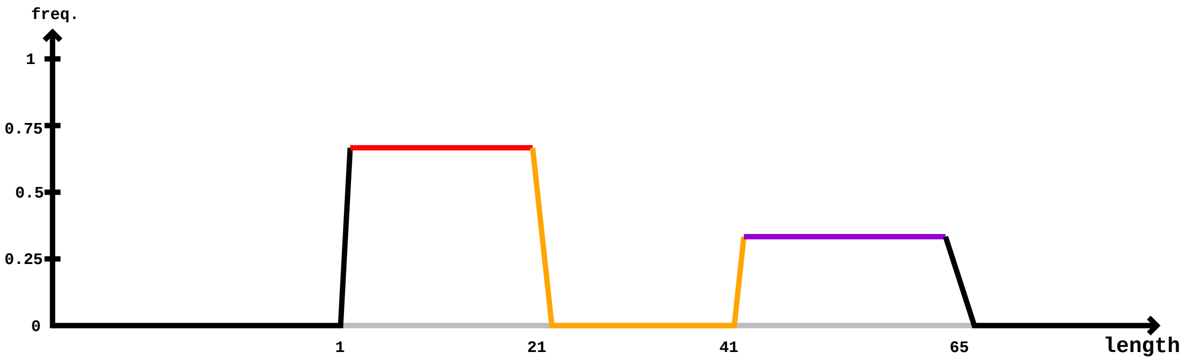

Mature Star

```
5'- ugagcuaugauuguggcacugcacugcagccugggcaacagagugagacccugugucuuaaagcaaaaucucacucuuuucguccaggcuggagugcaauggcgcgaucucgg  -3'  obs
    ugagcuaugauuguggcacugcacugcagccugggcaacagagugagacccugugucuuaaagcaaaaucucacucuuuucguccaggcuggagugcaauggcgcgaucucgg       exp
    ....((..(((((((.((.((((((.(((((((((((.(((((((((...(((........)))...))))))))).)).)))))))))).)))))).)).)))))))..))   reads   mm   sample
    .............................Ucugggcaacagagugagac............................................   1   1   seq
    .............................ccugggcaacaAagugagacc...........................................   1   1   seq
    ......................................................................ucacucuuuucguccaggcugga....................   1   0   seq
```

Provisional ID	:	chr17_2210
Score total	:	5.1
Score for star read(s)	:	3.9
Score for read counts	:	-4.9
Score for mfe	:	1.6
Score for randfold	:	1.6
Score for cons. seed	:	3
Total read count	:	2
Mature read count	:	1
Loop read count	:	0
Star read count	:	1

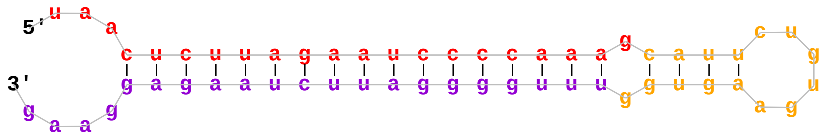

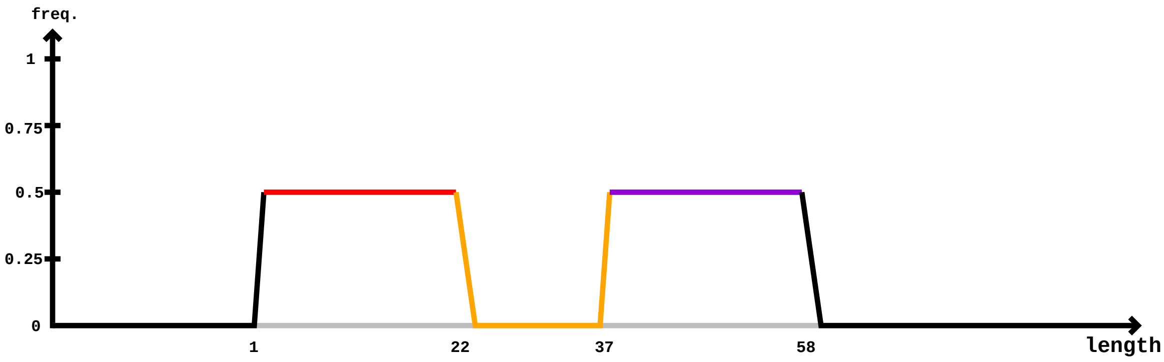

Mature Star

```
5'- cacaaagaacuguacugccuuaacucuuagaauccccaaagcauucugugaaguggGuuuggggauucuaagaggaagaagaguaaugucauuaauccauucuacagaugcg   -3'    obs
```

5'- cacaaagaacuguacugccuuaacucuuagaauccccaaagcauucugugaaguggguuuggggauucuaagaggaagaagaguaaugucauuaauccauucuacagaugcg	-3'	obs		
cacaaagaacuguacugccuuaacucuuagaauccccaaagcauucugugaaguggguuuggggauucuaagaggaagaagaguaaugucauuaauccauucuacagaugcg		exp		
........((..((((..(((..((((((((((((((((.((((......)))).))))))))))))))))).)))...))))..))........((((.....))))..	reads		mm	sample
....................uaacucuuagaauccccaaag..	1		0	seq
....uuuggggauucuaagaggaag..................................	1		0	seq

Provisional ID	:	chr22_2761
Score total	:	6.3
Score for star read(s)	:	3.9
Score for read counts	:	-1.3
Score for mfe	:	2.8
Score for randfold	:	1.6
Score for cons. seed	:	-0.6
Total read count	:	9
Mature read count	:	6
Loop read count	:	0
Star read count	:	3

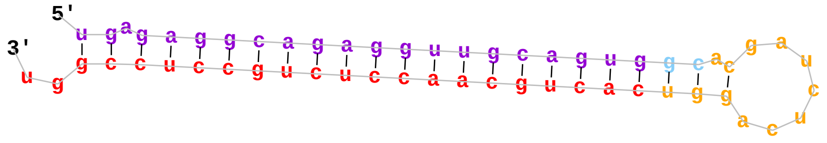

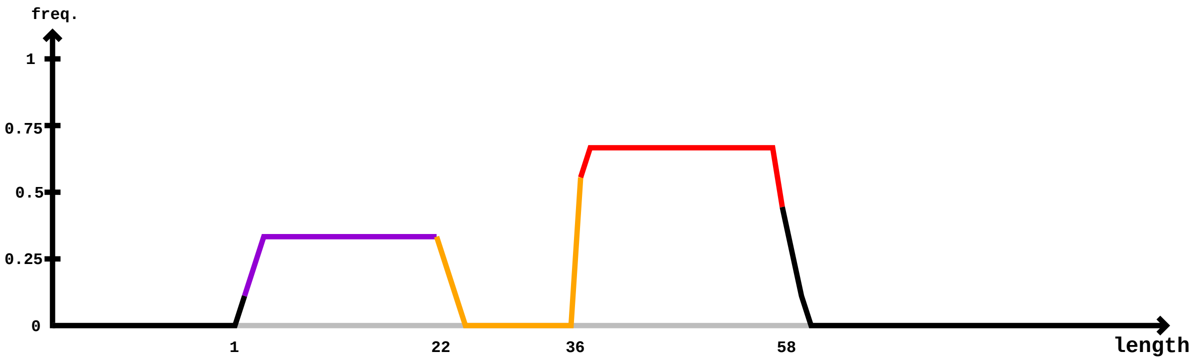


```
                    Star                            Mature
5'- ggagaaucgcauugaaccugagaggcagagguugcaguggcacgaucucaggucacugcaaccucugccuccgguauucaagcgauucuccugccucagccuuuugaggugaca   -3'    obs
    ggagaaucgcauugaaccugagaggcagagguugcaguggcacgaucucaggucacugcaaccucugccuccgguauucaagcgauucuccugccucagccuuuugaggugaca          exp
    ((((((((((.(((((.(((.((((((((((((((((((((.(.......)))))))))))))))))))))))))..)))))))))))))).(((((((....))))))).... reads  mm  sample
    ..................ugagaggcagagguugcagug...........................................................................  1     0   seq
    ...................gagaggcagagguugcagugg..........................................................................  1     0   seq
    ....................agaggcagagguugcaguggU.........................................................................  1     1   seq
    .....................................................cacugcaaccucugccuccgA........................................  1     1   seq
    .....................................................cacugcaaccucugccuccgg........................................  1     0   seq
    .....................................................cacugcaaccucugccuccggA.......................................  2     1   seq
    .....................................................cacugcaaccucugccuccggAa......................................  1     1   seq
    ......................................................acugcaaccucugccuccgguau.....................................  1     0   seq
```

Provisional ID	:	chr10_1053
Score total	:	17
Score for star read(s)	:	3.9
Score for read counts	:	18.5
Score for mfe	:	-2.4
Score for randfold	:	-2.2
Score for cons. seed	:	-0.6
Total read count	:	48
Mature read count	:	40
Loop read count	:	0
Star read count	:	8

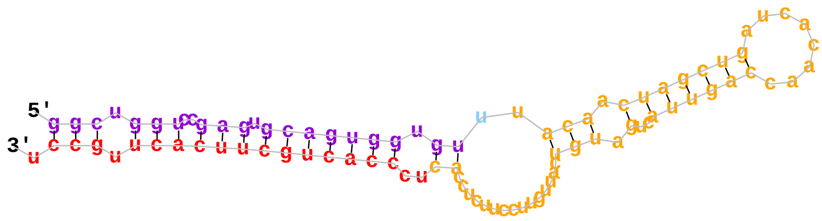

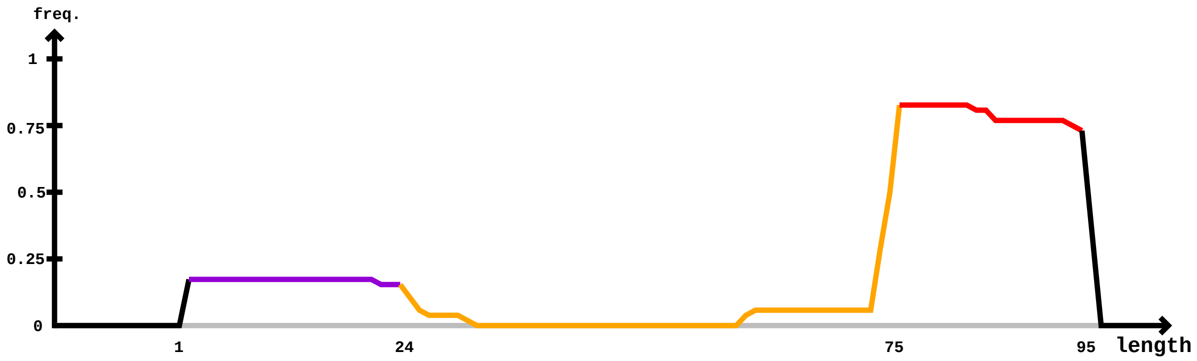

Star Mature

```
5'- acaguucacauaggcugguccgagugcagugguguuuacaacuagcugaucacaaccaguuacugaugucauuguuccuucuccacucccacugcuucacuugccuuuacaaaa   -3'    obs
    acaguucacauaggcugguccgagugcagugguguuuacaacuagcugaucacaaccaguuacugaugucauuguuccuucuccacucccacugcuucacuugccuuuacaaaa          exp
    ...........((((.(((..(((.(((((((.((..(((.(((((((........)))))))..).)))...............))..)))))))))))).))))........   reads     mm      sample
    ............ggcugguccgagugcagugg..................................................................................   1         0       seq
    ............ggcugguccgagugcaguggugu...............................................................................   3         0       seq
    ............ggcugguccgagugcagugguguu..............................................................................   2         0       seq
    ............ggcugguccgagugcagugguguuu.............................................................................   1         0       seq
    ............ggcugguccgagugcagugguguuuacaa.........................................................................   1         0       seq
    ............ggcugguccgagugcagugguguuuacaac........................................................................   1         0       seq
    ......................................................................auuguuccuucuccacucccacug....................   1         0       seq
    ......................................................................auugAuccuucuccacucccacugcu..................   1         1       seq
    .......................................................................uuguuccuucuccacucccacugcu..................   1         0       seq
    ....................................................................................acucccacugcuucacuugA..........   1         1       seq
    ....................................................................................acucccacugcuucacuugAc.........   1         1       seq
    ....................................................................................acucccacugcuucacuugAcu........   10        1       seq
    .....................................................................................cucccacugcuucacuugAcu........   11        1       seq
    ......................................................................................ucccacugcuucacuugAcu........   17        1       seq
```

Provisional ID	:	chr1_263
Score total	:	10.9
Score for star read(s)	:	3.9
Score for read counts	:	8.8
Score for mfe	:	-2.5
Score for randfold	:	-2.2
Score for cons. seed	:	3
Total read count	:	29
Mature read count	:	28
Loop read count	:	0
Star read count	:	1

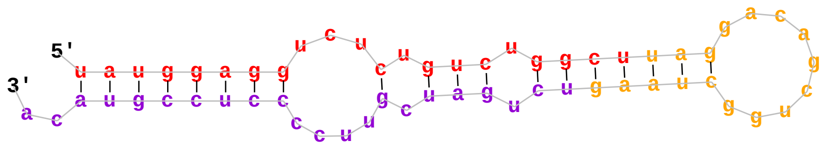

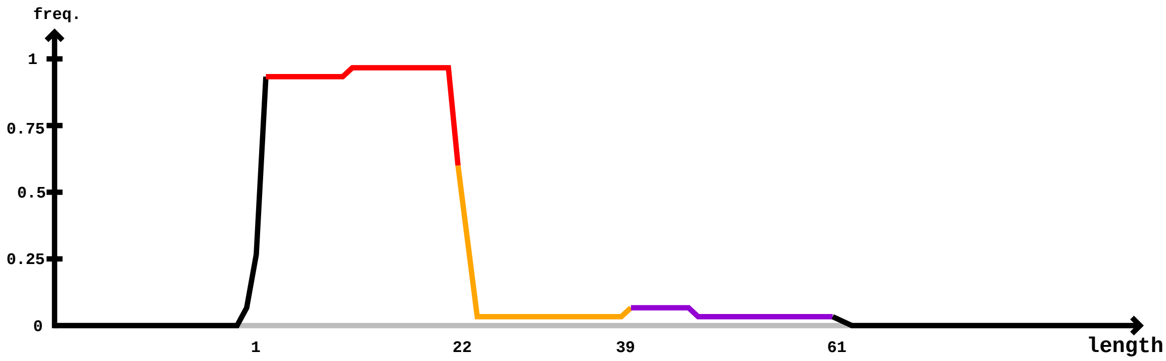

Mature **Star**

```
5'- gcagcgauccuacauaaauauauggaggucucugucuggcuuaggacagcuggcuaagucugaucguuccccuccguacagccuuuaaaacugccacucuaaauacuuggu   -3'   obs
    gcagcgauccuacauaaauauauggaggucucugucuggcuuaggacagcuggcuaagucugaucguuccccuccguacagccuuuaaaacugccacucuaaauacuuggu         exp
    ....................((((((((...(.(((.(((((((.........))))))).))).)....))))))))..............((((...........))))   reads   mm   sample
    ..................uauauggaggucucugucuggc.......................................................................   2       0    seq
    ...................auauggaggucGcugucuggc.......................................................................   1       1    seq
    ...................auauggaggucucugucuggc.......................................................................   2       0    seq
    ...................auauggaggucucugucuggcu......................................................................   1       0    seq
    ...................auauggaggucucugucuggcA......................................................................   2       1    seq
    ....................uauggaggucucugucuggc.......................................................................   6       0    seq
    ....................uauggaggucucugucuggcu......................................................................   13      0    seq
    ....................uauggaggucucugucuggcuu.....................................................................   1       0    seq
    .............................cucugucuggcuuaggacagcuggcuaagucugauc..............................................   1       0    seq
    ..........................................................ucugaucguuccccuccAuaca...............................   1       1    seq
```

Provisional ID	:	chr2_396
Score total	:	5.4
Score for star read(s)	:	-1.3
Score for read counts	:	0
Score for mfe	:	2.1
Score for randfold	:	1.6
Score for cons. seed	:	3
Total read count	:	13
Mature read count	:	13
Loop read count	:	0
Star read count	:	0

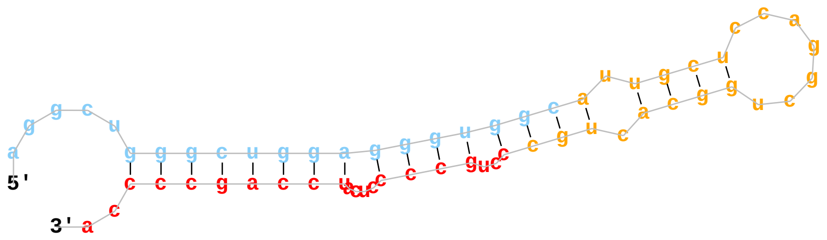

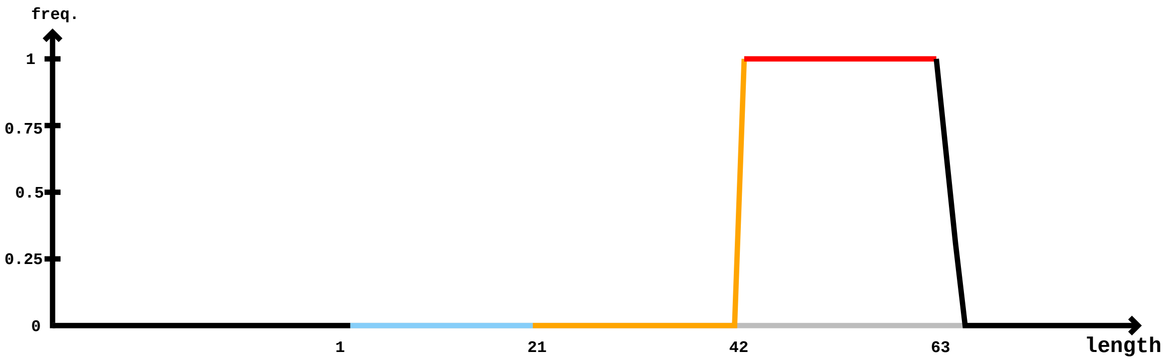

Star **Mature**

```
5'- cagcuuagggagagguggugcgaccagugaggcugggcuggaggguggcauugcuccaggcuggcacugcccugccccucauccagccccacugcaucccugaagggcagu   -3'    exp
    ..((((......(((.(((((....((((.(((((((.(((.((((((((.((((.......)))).))))..)))).)))))))))).))))))))))))...))))...    reads    mm    sample
    ......................................................................ccugccccucauccagcccca....................    7        0     seq
    ......................................................................ccugccccucauccagccccac...................    1        0     seq
    ......................................................................ccugccccucauccagccccaU...................    1        1     seq
    ......................................................................ccugccccucauccagccccacu..................    4        0     seq
```

Provisional ID	:	chr6_872
Score total	:	5.2
Score for star read(s)	:	-1.3
Score for read counts	:	0
Score for mfe	:	1.9
Score for randfold	:	1.6
Score for cons. seed	:	3
Total read count	:	15
Mature read count	:	15
Loop read count	:	0
Star read count	:	0

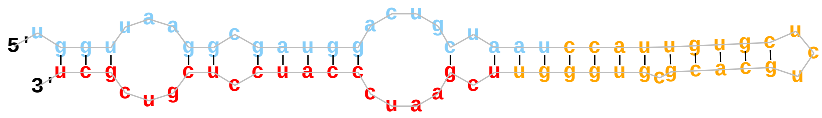

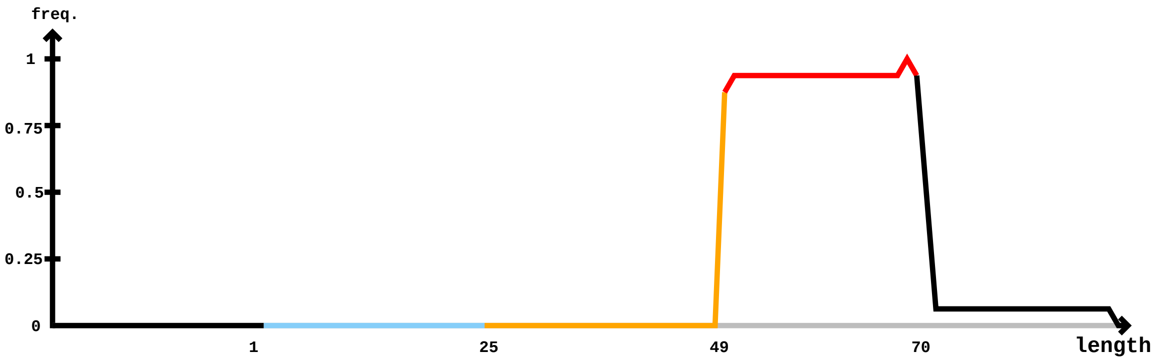

Star Mature

```
5'- ucaggagaggccuggccgagugguuaaggcgauggacugcuaauccauugugcucugcacgcguggguucgaaucccauccucgucgcucggcuuucccugcuaacuggg   -3'   exp
    ((((.(((((...((((((((((....((.(((((....(.((((((((((((...))))).))))))).)....)))))))..))))))))))...))).))..)))).   reads   mm   sample
    ....................................................................ucgaaucccauccucgucgc......................   1       0    seq
    ....................................................................ucgaaucccauccucgucgcC.....................   13      1    seq
    .....................................................................cgaaucccauccucgucgcC.....................   1       1    seq
    .......................................................................................cucggcuuucccugcuaacugg.   1       0    seq
```

Provisional ID	:	chr7_1027
Score total	:	5.5
Score for star read(s)	:	-1.3
Score for read counts	:	0
Score for mfe	:	2.2
Score for randfold	:	1.6
Score for cons. seed	:	3
Total read count	:	13
Mature read count	:	13
Loop read count	:	0
Star read count	:	0

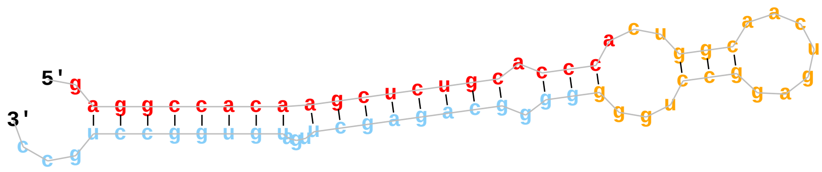

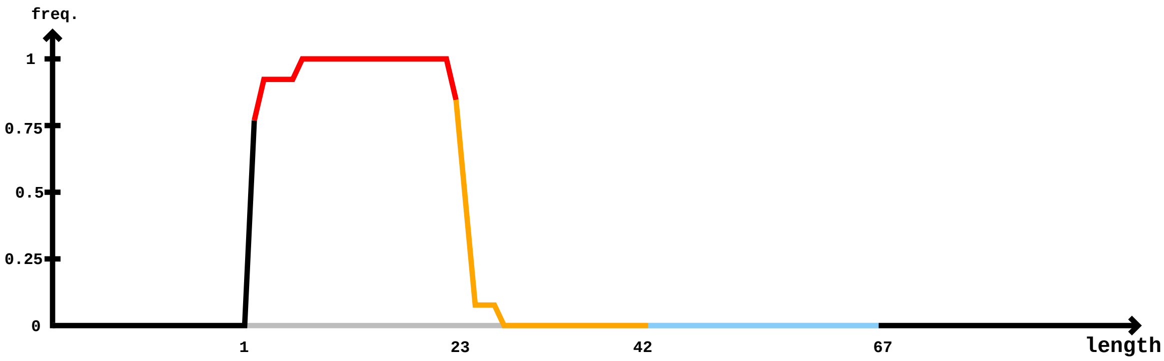

Mature   Star

```
5'- cuacccagcagaacucccugaggccacaagcucugcacccacuggcaacugaggccugggggggcagagcuugauguggccugccugagcucugucccguacaucccccgc   -3'    exp
    .(((...(((((.(((....((((((((((((((.(((...(((.......)))...))).))))))))...))))))))....))).)))))...)))..........   reads        mm          sample
    ...................gaggccacaagcucugcaccc.......................................................................   2            0           seq
    ...................gaggccacaagcucugcaccca......................................................................   6            0           seq
    ...................gaggccacaagcucuUcaccca......................................................................   1            1           seq
    ...................gaggccacaagcucugcacccU......................................................................   1            1           seq
    ....................aggccacaagcucugcaccca......................................................................   1            0           seq
    ....................aggccacaCgcucugcaccca......................................................................   1            1           seq
    ........................cacaagcucugcacccacugg..................................................................   1            0           seq
```

Provisional ID	:	chr12_1771
Score total	:	5.8
Score for star read(s)	:	3.9
Score for read counts	:	-0.8
Score for mfe	:	1.7
Score for randfold	:	1.6
Score for cons. seed	:	-0.6
Total read count	:	10
Mature read count	:	9
Loop read count	:	0
Star read count	:	1

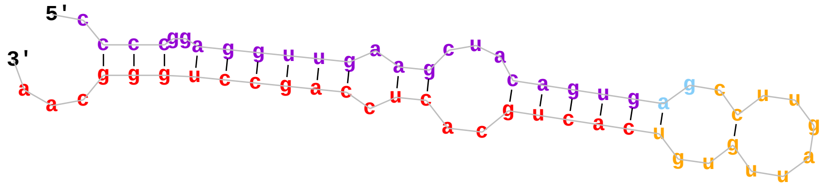

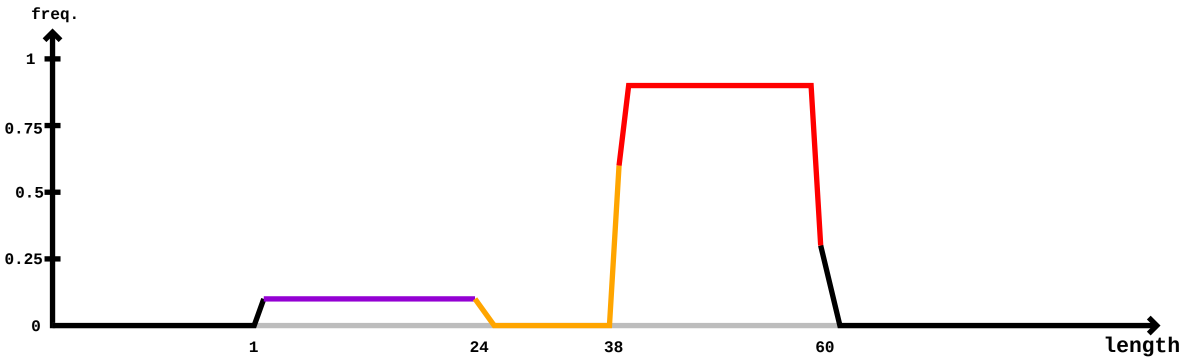

Star **Mature**

```
5'- aggugggaggauugcuuguuccccggagguugaagcuacagugagccuugauuguguacacugcacuccagccugggcaacagguaagacucugucucaaaaaaaaacaaaaaa   -3'    obs
    aggugggaggauugcuuguuccccggagguugaagcuacagugagccuugauugugucacugcacuccagccugggcaacagguaagacucugucucaaaaaaaaacaaaaaa          exp
    .((..((((..(((((((((.(((..((((((.((...((((((..(......)..))))))..)).))))))))).)))))))))..))))..)).................   reads     mm     sample
    ....................ccccggagguugaagcuacagug......................................................................   1         0      seq
    .........................................................cacugcacuccagccugggca...................................   2         0      seq
    .........................................................caUugcacuccagccugggca...................................   1         1      seq
    .........................................................cacugcacuccagccugggcaa..................................   1         0      seq
    .........................................................cacugcacuccagccugggcGa..................................   2         1      seq
    ..........................................................acugcacuccUgccugggca...................................   1         1      seq
    ..........................................................acugcacuccagccugggca...................................   2         0      seq
```

Provisional ID	:	chr1_257
Score total	:	9.3
Score for star read(s)	:	3.9
Score for read counts	:	7.3
Score for mfe	:	-2.5
Score for randfold	:	-2.2
Score for cons. seed	:	3
Total read count	:	26
Mature read count	:	18
Loop read count	:	0
Star read count	:	8

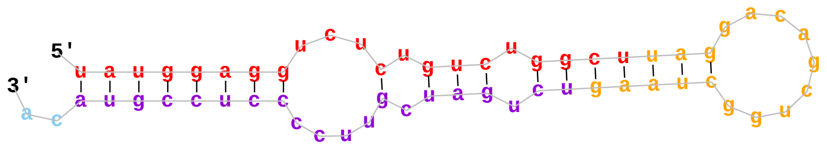

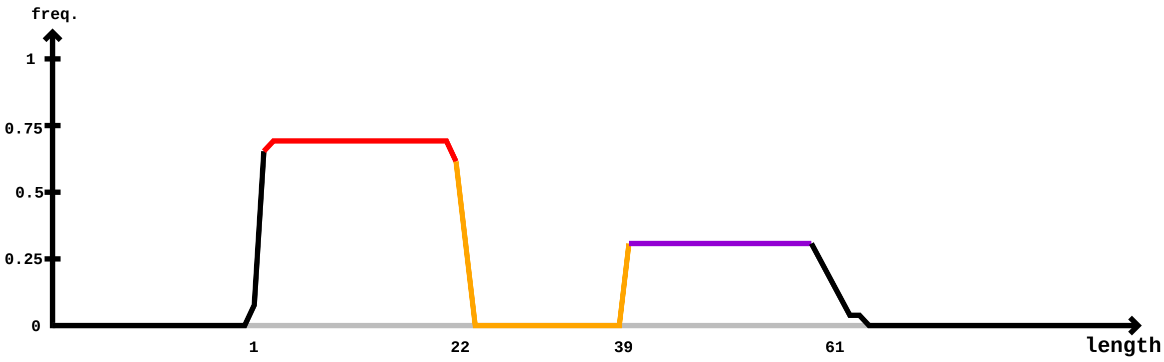


```
                          Mature                               Star
5'- gcagcgauccuacauaaauauauggaggucucugucuggcuuaggacagcuggcuaagucugaucguuccccuccguacagccuuuaaaacugccacucuaaauacuuggu   -3'    obs
    gcagcgauccuacauaaauauauggaggucucugucuggcuuaggacagcuggcuaagucugaucguuccccuccguacagccuuuaaaacugccacucuaaauacuuggu          exp
    ....................((((((((...(.(((.(((((((.........))))))).))).)....))))))))..............((((...........))))   reads      mm       sample
    ...................Cuauggaggucucugucuggcu......................................................................   1          1        seq
    ...................auauggaggucucugucuggcA......................................................................   1          1        seq
    ....................uauggaggucucugucuggc.......................................................................   2          0        seq
    ....................uauggaggucucugucuggcu......................................................................   13         0        seq
    .....................auggaggucucugucuggcu......................................................................   1          0        seq
    ..........................................................ucugaucguuccccuccAua.................................   6          1        seq
    ..........................................................ucugaucguuccccuccAuacag..............................   1          1        seq
    ..........................................................ucugaucguuccccuccAuacagcc............................   1          1        seq
```

Provisional ID	:	chr3_592
Score total	:	5.7
Score for star read(s)	:	3.9
Score for read counts	:	-4.9
Score for mfe	:	2.2
Score for randfold	:	1.6
Score for cons. seed	:	3
Total read count	:	2
Mature read count	:	1
Loop read count	:	0
Star read count	:	1

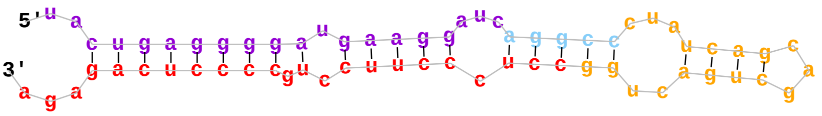

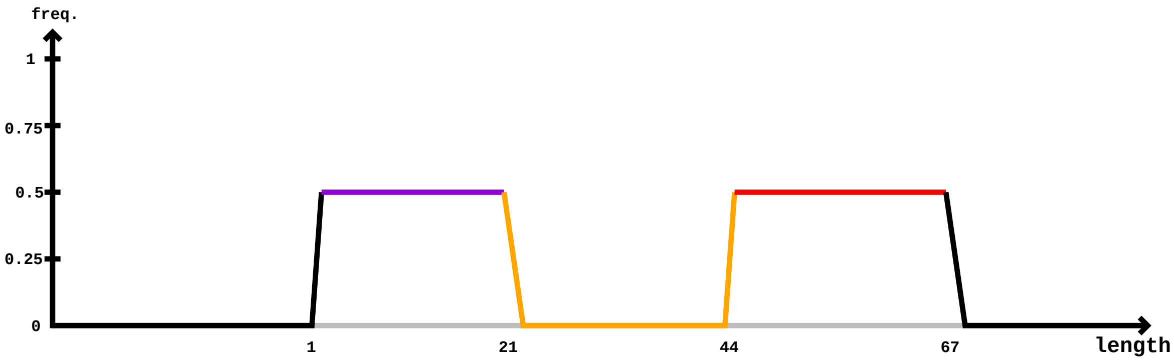

Star Mature

```
5'- aaacugucccuggcugagccccaccuuacugaggggaugaaggaucaggcccuaucagcagcugacuggccucccuuccugccccucagagaacagugaggccccagaggag  -3'  obs
    aaacugucccuggcugagccccaccuuacugaggggaugaaggaucaggcccuaucagcagcugacuggccucccuuccugccccucagagaacagugaggccccagaggag       exp
    ......(((((((..(.(((.(((.((.(((((((((.(((((...(((((...((((...))))..))))).))))).).)))))))).))...))).)))))))).))).   reads  mm  sample
    ..........................uacugaggggaugaaggauc..................................................................   1      0   seq
    .....................................................................ccucccuuccugccccucagagU....................   1      1   seq
```

Provisional ID	:	chr7_1125
Score total	:	5.5
Score for star read(s)	:	-1.3
Score for read counts	:	0
Score for mfe	:	2.2
Score for randfold	:	1.6
Score for cons. seed	:	3
Total read count	:	14
Mature read count	:	14
Loop read count	:	0
Star read count	:	0

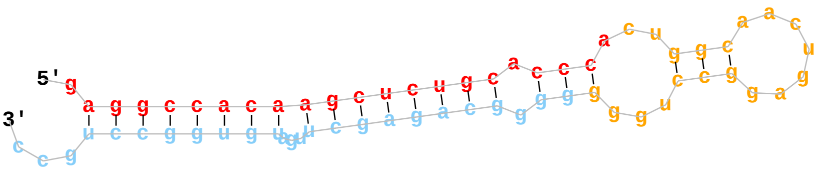

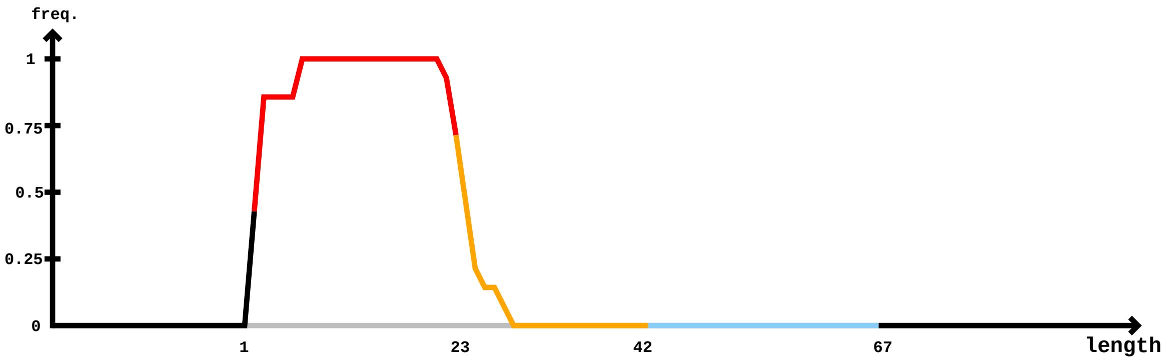

Mature Star

```
5'- cuacccagcagaacucccugaggccacaagcucugcacccacuggcaacugaggccuggggggggcagagcuugauguggccugccugagcucugucccguacaucccccgc   -3'    exp
    .(((...(((((.(((....((((((((((((((((.(((...(((.......)))...))).))))))))...))))))))....))).)))))...)))..........    reads      mm        sample
    ...................gaggccacaagcucugcacc........................................................................    1          0         seq
    ...................gaggccacaagcucugcaccc.......................................................................    1          0         seq
    ...................gaggccacaagcucugcaccca......................................................................    3          0         seq
    ...................gaggccacaagcucugcacccU......................................................................    1          1         seq
    ....................aggccacaagcucugcaccc.......................................................................    1          0         seq
    ....................aggccacaagcucugcaccU.......................................................................    1          1         seq
    ....................aggccacaagcucugcaccca......................................................................    3          0         seq
    ....................aggccacaagcucugcacccacu....................................................................    1          0         seq
    ........................cacaagcucugcacccacugg..................................................................    1          0         seq
    ........................cacaagcucugcacccacuggc.................................................................    1          0         seq
```

Provisional ID	:	chr22_2922
Score total	:	5.5
Score for star read(s)	:	-1.3
Score for read counts	:	0
Score for mfe	:	2.2
Score for randfold	:	1.6
Score for cons. seed	:	3
Total read count	:	15
Mature read count	:	15
Loop read count	:	0
Star read count	:	0

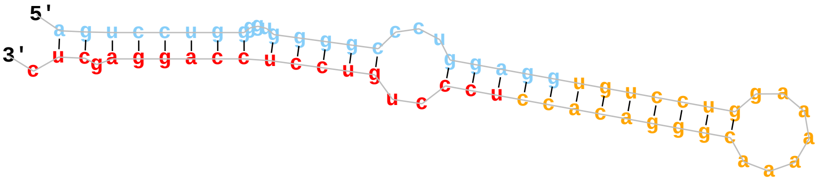

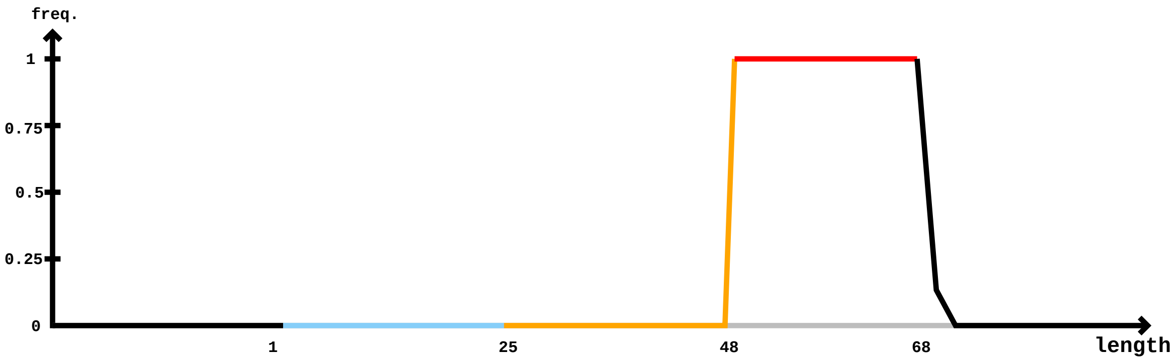

Star Mature

```
5'- cugcgugucuggcacagagcgaaguccugggguggggcccuggagguguccuggaaaaacgggacaccucccuguccuccaggagcucacccuaagucacagaagcccaug   -3'    exp
    .((.((.((((..((((.(.((..((((((...(((((...(((((((((((.......)))))))))))..)))))))))))..)).).))..))..)))).)).))..    reads      mm          sample
    ..................................................................ucccuguccuccaggagcuc......................    8          0           seq
    ..................................................................ucccuguccuccaggagcuG......................    1          1           seq
    ..................................................................ucccuguccuccaggagcuca.....................    2          0           seq
    ..................................................................ucccuguccuccaggagcucU.....................    2          1           seq
    ..................................................................ucccuguccuccaggagcucaU....................    1          1           seq
    ..................................................................ucccuguccuccaggagcucacA...................    1          1           seq
```

Provisional ID	:	chr1_245
Score total	:	13.9
Score for star read(s)	:	3.9
Score for read counts	:	11.9
Score for mfe	:	-2.5
Score for randfold	:	-2.2
Score for cons. seed	:	3
Total read count	:	35
Mature read count	:	32
Loop read count	:	0
Star read count	:	3

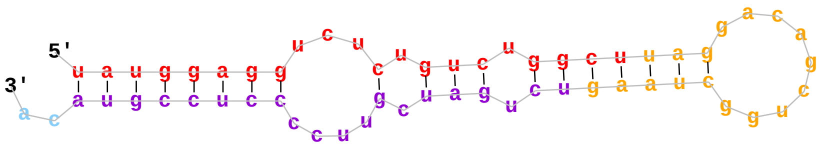

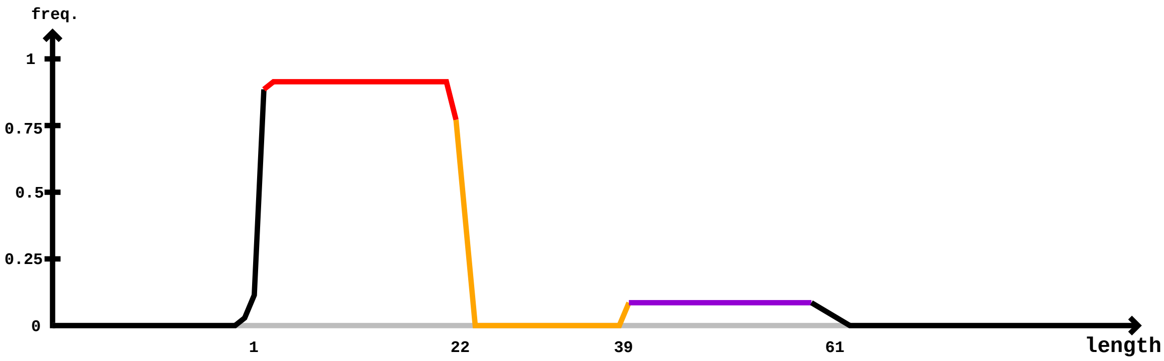

Mature Star

```
5'- gcagcgauccuacauaaauauauggaggucucugucuggcuuaggacagcuggcuaagucugaucguuccccuccguacagccuuuaaaacugccacucuaaauacuuggu   -3'   obs
    gcagcgauccuacauaaauauauggaggucucugucuggcuuaggacagcuggcuaagucugaucguuccccuccguacagccuuuaaaacugccacucuaaauacuuggu         exp
    ....................((((((((...(.(((.(((((((.........))))))).))).)....))))))))..............((((...........))))   reads   mm   sample
    ..................uauauggaggucucugucuggcu......................................................................   1       0    seq
    ...................auauggaggucucugucuggcu......................................................................   2       0    seq
    ...................auauggaggucucugucuggcA......................................................................   1       1    seq
    ....................uauggaggucucugucuggc.......................................................................   5       0    seq
    ....................uauggaggucucugucuggcu......................................................................   21      0    seq
    ....................uauggaggucucugucuggcA......................................................................   1       1    seq
    .....................auggaggucucugucuggcu......................................................................   1       0    seq
    ..........................................................ucugaucguuccccuccAua.................................   2       1    seq
    ..........................................................ucugaucguuccccuccAuaca...............................   1       1    seq
```

Provisional ID	:	chr17_2271
Score total	:	6.2
Score for star read(s)	:	-1.3
Score for read counts	:	0
Score for mfe	:	2.9
Score for randfold	:	1.6
Score for cons. seed	:	3
Total read count	:	13
Mature read count	:	13
Loop read count	:	0
Star read count	:	0

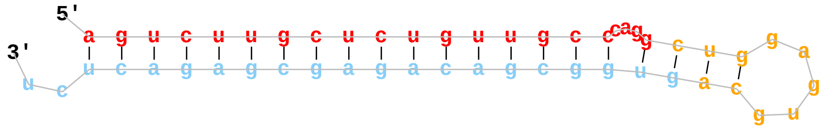

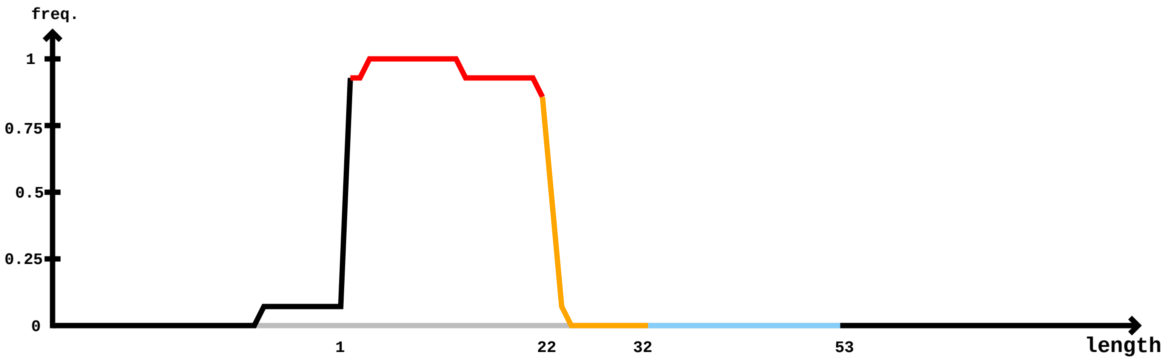

Mature **Star**

```
5'- ccauuucuauacuuuuuuuugcgagccagagucuugcucuguugcccaggcuggagugcaguggcgacagagcgagacucuggcucaaaaaaaaaaaaaguauagaaaugg   -3'    exp
    ((((((((((((((((((..((((((((((((((((((((((((((...((((.....))))))))))))))))))))))))))))))))).....))))))))))))))))))    reads       mm          sample
    ....................gcgagccagagucuugcucug......................................................................    1           0           seq
    .............................agucuugcucuguCgcccag..............................................................    1           1           seq
    .............................agucuugcucuguCgcccagg.............................................................    10          1           seq
    .............................agucuugcucuguCgcccaggc............................................................    1           1           seq
    ...............................ucuugcuGuguugcccaggcu...........................................................    1           1           seq
```

Provisional ID	:	chr22_2737
Score total	:	8.5
Score for star read(s)	:	3.9
Score for read counts	:	1.1
Score for mfe	:	2.5
Score for randfold	:	1.6
Score for cons. seed	:	-0.6
Total read count	:	14
Mature read count	:	8
Loop read count	:	0
Star read count	:	6

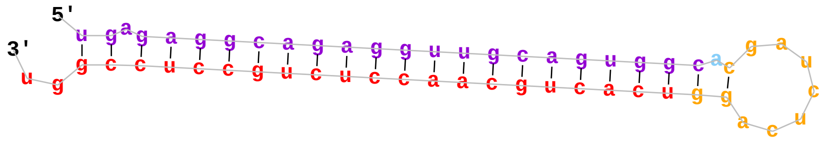

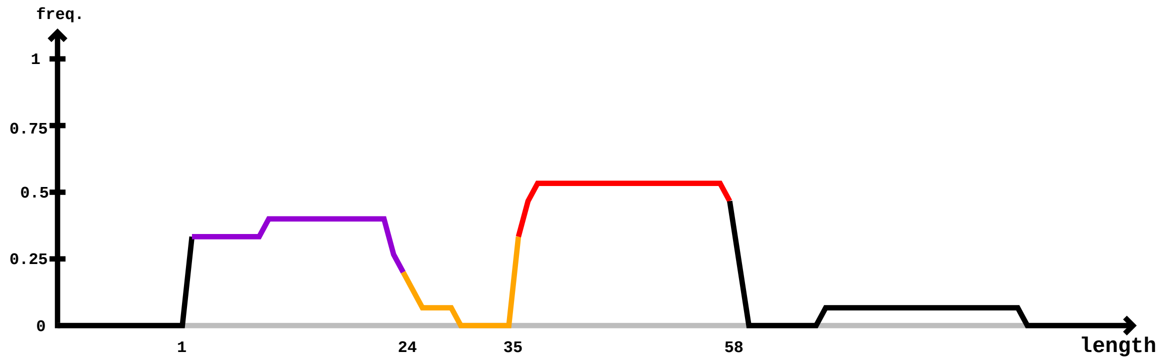


```
              Star                   Mature
5'- ucgcauugaaccugagaggcagagguugcaguggcacgaucucaggucacugcaaccucugccuccgguauucaagcgauucuccugccucagccuuuugaggugacaaa   -3'    obs
    ucgcauugaaccugagaggcagagguugcaguggcacgaucucaggucacugcaaccucugccuccgguauucaagcgauucuccugccucagccuuuugaggugacaaa          exp
    ((((.(((((.(((.((((((((((((((((((((.(.......))))))))))))))))))))))))..))))))))).......(((((((....)))))))......   reads      mm      sample
    ............ugagaggcagagguugcagug.............................................................................   2          0       seq
    ............ugagaggcagagguugcagugg............................................................................   1          0       seq
    ............ugagaggcagagguugcaguggU...........................................................................   1          1       seq
    ............ugagaggcagagguugcaguggc...........................................................................   1          0       seq
    ....................agagguugcaguggcacgau......................................................................   1          0       seq
    ..............................................ucacugcaaccucugccuccgg..........................................   1          0       seq
    ..............................................ucacugcaaccucugccuccggA.........................................   3          1       seq
    ..............................................ucacugcaaccucugccuccggAa........................................   1          1       seq
    ...............................................cacugcaaccucugccuccggu.........................................   1          0       seq
    ...............................................cacugcaaccucugccuccggAa........................................   1          1       seq
    ................................................acugcaaccucugccuccggua........................................   1          0       seq
    ..............................................................................auucuccugccucagccuuuu...........   1          0       seq
```

Provisional ID	:	chr1_117
Score total	:	7.3
Score for star read(s)	:	3.9
Score for read counts	:	5.2
Score for mfe	:	-2.5
Score for randfold	:	-2.2
Score for cons. seed	:	3
Total read count	:	22
Mature read count	:	21
Loop read count	:	0
Star read count	:	1

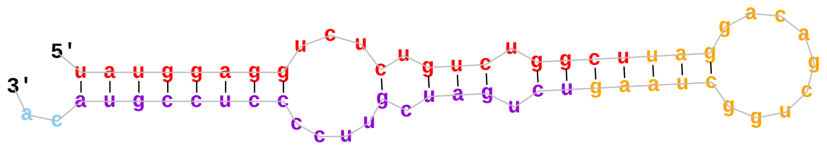

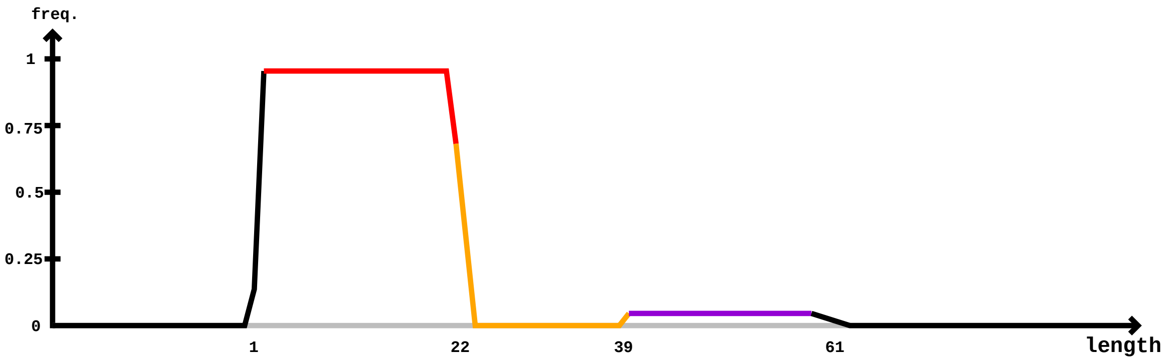

Mature **Star**

```
5'- gcagcgauccuacauaaauauauggaggucucugucuggcuuaggacagcuggcuaagucugaucguuccccuccguacagccuuuaaaacugccacucuaaauacuuggu   -3'    obs
    gcagcgauccuacauaaauauauggaggucucugucuggcuuaggacagcuggcuaagucugaucguuccccuccguacagccuuuaaaacugccacucuaaauacuuggu          exp
    ....................((((((((...(.(((.(((((((.........))))))).))).)....))))))))..............((((...........))))   reads     mm        sample
    ...................auauggaggucucugucuggc.......................................................................   2         0         seq
    ...................auauggaggucucugucuggcu......................................................................   1         0         seq
    ....................uauggaggucucugucuggc.......................................................................   4         0         seq
    ....................uauggaggucucugucuggcu......................................................................   14        0         seq
    ..........................................................ucugaucguuccccuccAua.................................   1         1         seq
```

Provisional ID	:	chr1_107
Score total	:	10.4
Score for star read(s)	:	3.9
Score for read counts	:	8.3
Score for mfe	:	-2.5
Score for randfold	:	-2.2
Score for cons. seed	:	3
Total read count	:	28
Mature read count	:	26
Loop read count	:	0
Star read count	:	2

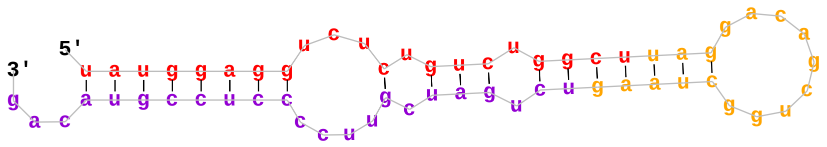

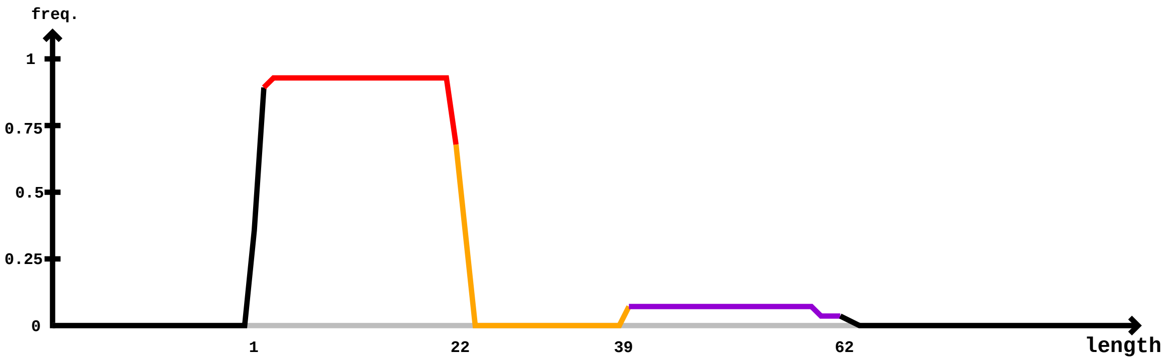

Mature Star

```
5'- gcagcgauccuacauaaauauauggaggucucugucuggcuuaggacagcuggcuaagucugaucguuccccuccguacagccuuuaaaacugccacucuaaauacuuggu   -3'   obs
    gcagcgauccuacauaaauauauggaggucucugucuggcuuaggacagcuggcuaagucugaucguuccccuccguacagccuuuaaaacugccacucuaaauacuuggu         exp
    ....................((((((((...(.(((.(((((((.........))))))).))).)....)))))))).............((((...........))))   reads   mm   sample
    ...................auauggaggucucugucuggc.......................................................................   3       0    seq
    ...................auauggaggucucugucuggcu......................................................................   7       0    seq
    ....................uauggaggucucugucuggc.......................................................................   4       0    seq
    ....................uauggaggucucugucuggcA......................................................................   1       1    seq
    ....................uauggaggucucugucuggcu......................................................................   10      0    seq
    .....................auggaggucucugucuggcu......................................................................   1       0    seq
    ..........................................................ucugaucguuccccuccAua.................................   1       1    seq
    ..........................................................ucugaucguuccccuccAuacag..............................   1       1    seq
```

Provisional ID	:	chr22_1399
Score total	:	6.5
Score for star read(s)	:	3.9
Score for read counts	:	-0.8
Score for mfe	:	2.5
Score for randfold	:	1.6
Score for cons. seed	:	-0.6
Total read count	:	10
Mature read count	:	4
Loop read count	:	0
Star read count	:	6

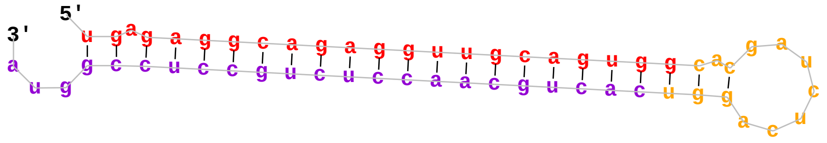

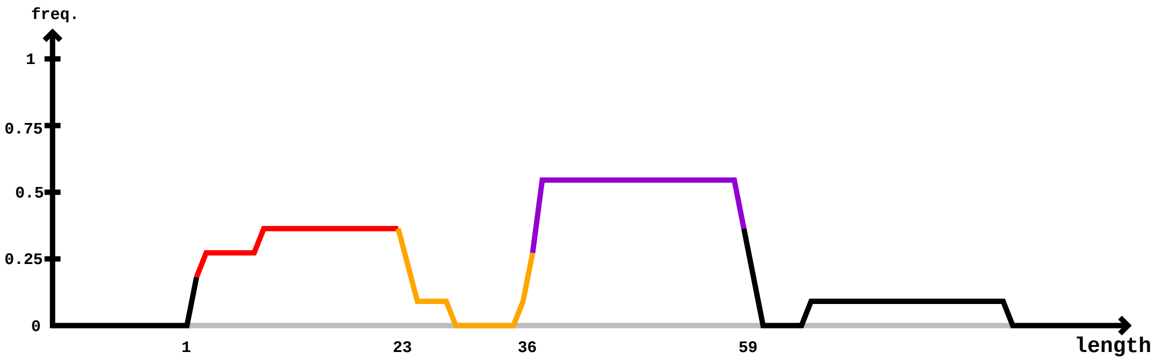


```
                 Mature                             Star
5'- aucgcauugaaccugagaggcagagguugcaguggcacgaucucaggucacugcaaccucugccuccgguauucaagcgauucuccugccucagccuuuugaggugacaa   -3'   obs
    aucgcauugaaccugagaggcagagguugcaguggcacgaucucaggucacugcaaccucugccuccgguauucaagcgauucuccugccucagccuuuugaggugacaa         exp
    (((((.(((((.(((.((((((((((((((((((((.(.......))))))))))))))))))))))))..))))))))))......(((((((....))))))).....   reads    mm    sample
    .............ugagaggcagagguugcagugg...........................................................................   2        0     seq
    ..............gagaggcagagguugcagugg...........................................................................   1        0     seq
    ....................cagagguugcaguggcacga......................................................................   1        0     seq
    ...............................................ucacugcaaccucugccuccggA........................................   1        1     seq
    ................................................cacugcaaccucugccuccggAa.......................................   1        1     seq
    ................................................cacugcaaccucugccuccggua.......................................   1        0     seq
    .................................................acugcaaccucugccuccggu........................................   1        0     seq
    .................................................acugcaaccucugccuccggAa.......................................   1        1     seq
    .................................................acugcaaccucugccuccggua.......................................   1        0     seq
    .............................................................................cgauucuccugccucagccuu............   1        0     seq
```

Provisional ID	:	chr1_99
Score total	:	5.8
Score for star read(s)	:	3.9
Score for read counts	:	3.7
Score for mfe	:	-2.5
Score for randfold	:	-2.2
Score for cons. seed	:	3
Total read count	:	19
Mature read count	:	17
Loop read count	:	0
Star read count	:	2

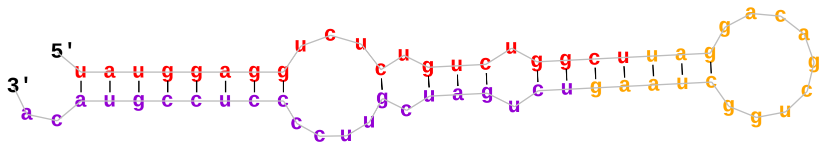

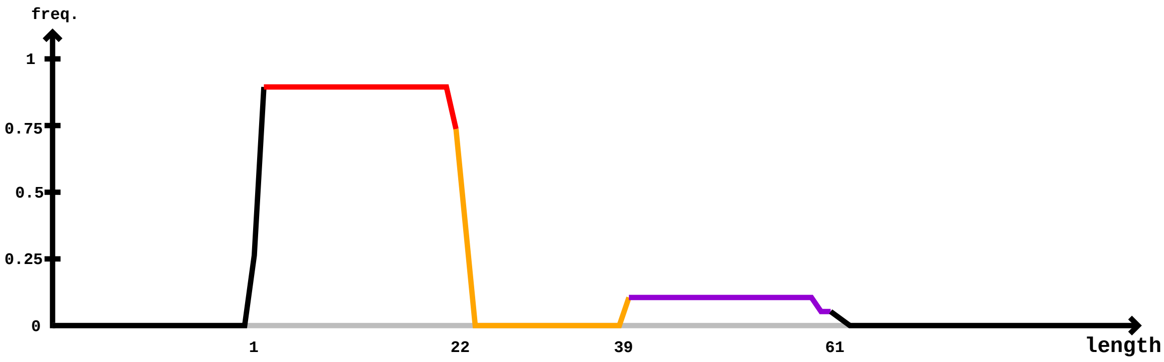

Mature Star

```
5'- gcagcgauccuacauaaauauauggaggucucugucuggcuuaggacagcuggcuaagucugaucguuccccuccguacagccuuuaaaacugccacucuaaauacuuggu   -3'    obs
    gcagcgauccuacauaaauauauggaggucucugucuggcuuaggacagcuggcuaagucugaucguuccccuccguacagccuuuaaaacugccacucuaaauacuuggu          exp
    ....................((((((((...(.(((.(((((((.........))))))).))).)....))))))))..............((((...........))))    reads      mm        sample
    ...................auauggaggucucugucuggc.......................................................................    2          0         seq
    ...................auauggaggucucugucuggcu......................................................................    3          0         seq
    ....................uauggaggucucugucuggc.......................................................................    1          0         seq
    ....................uCuggaggucucugucuggcu......................................................................    2          1         seq
    ....................uauggaggucucugucuggcu......................................................................    8          0         seq
    ....................uauggaggucucugucuggcA......................................................................    1          1         seq
    ..........................................................ucugaucguuccccuccgua.................................    1          0         seq
    ..........................................................ucugaucguuccccuccguacU...............................    1          1         seq
```

Provisional ID	:	chr6_356
Score total	:	5.2
Score for star read(s)	:	-1.3
Score for read counts	:	0
Score for mfe	:	1.9
Score for randfold	:	1.6
Score for cons. seed	:	3
Total read count	:	22
Mature read count	:	22
Loop read count	:	0
Star read count	:	0

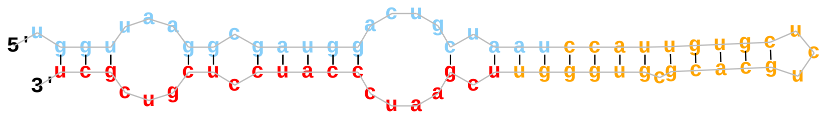

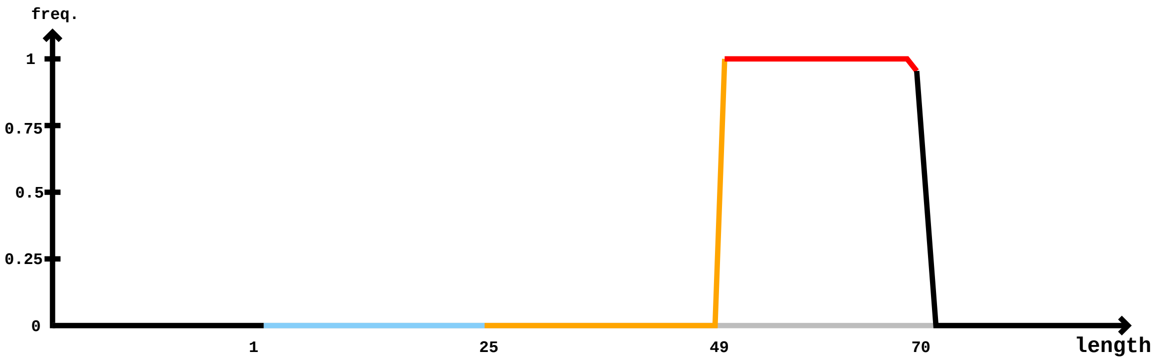

Star Mature

```
5'- ucaggagaggccuggccgagugguuaaggcgauggacugcuaauccauugugcucugcacgcgugggguucgaaucccauccucgucgcucggcuuucccugcuaacuggg   -3'    exp
    ((((.(((((...((((((((((....((.(((((....(.(((((((((((((...))))).))))))).)....)))))))..))))))))))...))).))..)))).   reads      mm          sample
    ....................................................................ucgaaucccauccucgucgc......................   1          0           seq
    ....................................................................ucgaaucccauccucgucgcC.....................   21         1           seq
```

Provisional ID	:	chr1_193
Score total	:	7.8
Score for star read(s)	:	3.9
Score for read counts	:	5.7
Score for mfe	:	-2.5
Score for randfold	:	-2.2
Score for cons. seed	:	3
Total read count	:	23
Mature read count	:	19
Loop read count	:	0
Star read count	:	4

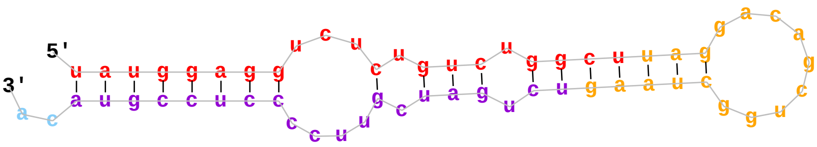

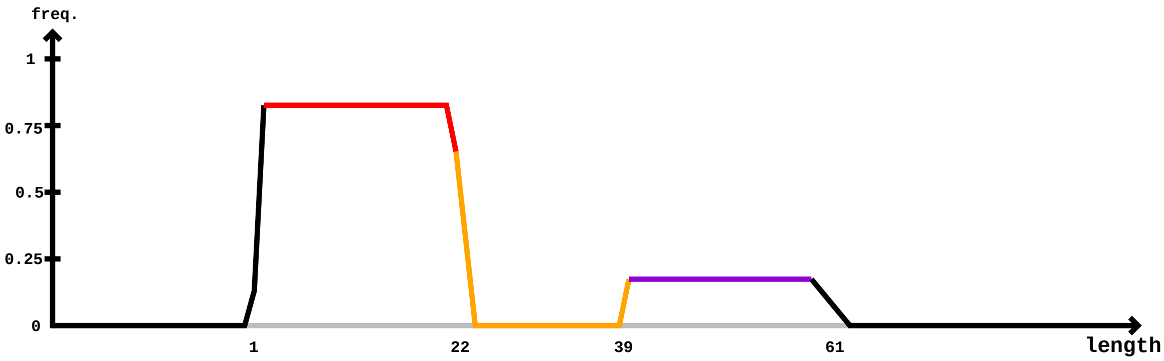

Mature Star

```
5'- gcagcgauccuacauaaauauauggaggucucugucuggcuuaggacagcuggcuaagucugaucguuccccuccguacagccuuuaaaacugccacucuaaauacuuggu  -3'  obs
    gcagcgauccuacauaaauauauggaggucucugucuggcuuaggacagcuggcuaagucugaucguuccccuccguacagccuuuaaaacugccacucuaaauacuuggu        exp
    ....................((((((((...(.(((.(((((((.........))))))).))).)....)))))))).............((((............))))  reads  mm  sample
    ..................auauggaggucucugucuggc........................................................................  1      0   seq
    ..................auauggaggucucugucuggcu.......................................................................  2      0   seq
    ....................uauggaggucucugucuggc.......................................................................  3      0   seq
    ....................uCuggaggucucugucuggcu......................................................................  1      1   seq
    ....................uauggaggucucugucuggcu......................................................................  12     0   seq
    ..........................................................ucugaucguuccccuccAua.................................  4      1   seq
```

Provisional ID	:	chr1_167
Score total	:	6.8
Score for star read(s)	:	3.9
Score for read counts	:	4.7
Score for mfe	:	-2.5
Score for randfold	:	-2.2
Score for cons. seed	:	3
Total read count	:	21
Mature read count	:	12
Loop read count	:	0
Star read count	:	9

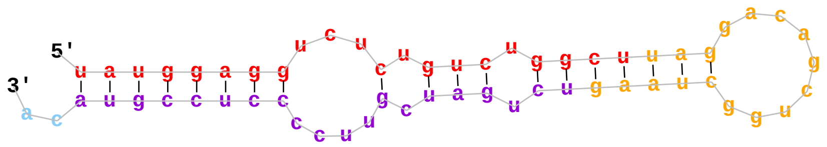

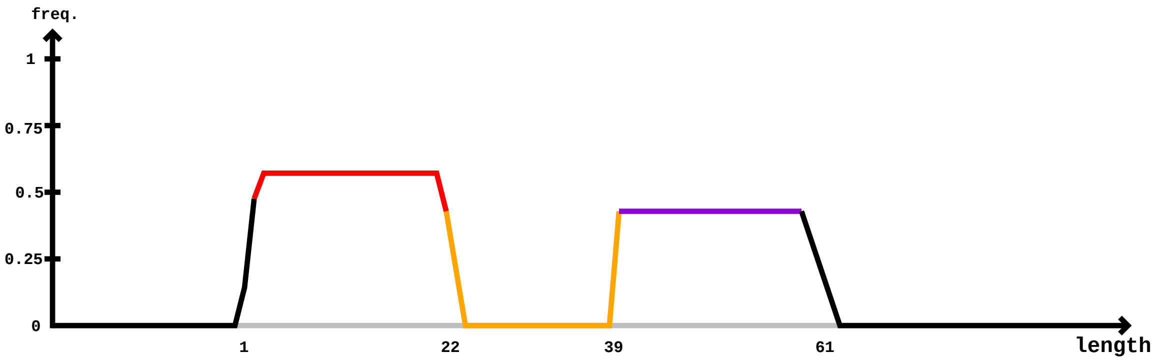

Mature Star

```
5'- cagcgauccuacauaaauauauggaggucucugucuggcuuaggacagcuggcuaagucugaucguuccccuccguacagccuuuaaaacugccacucuaaauacuuggu   -3'    obs
    cagcgauccuacauaaauauauggaggucucugucuggcuuaggacagcuggcuaagucugaucguuccccuccguacagccuuuaaaacugccacucuaaauacuuggu          exp
    ...................((((((((...(.(((.(((((((.........))))))).))).)....))))))))..............((((...........))))   reads    mm    sample
    ..................auauggaggucucugucuggc.......................................................................   2        0     seq
    ..................auauggaggucucugucuggcu......................................................................   1        0     seq
    ...................uauggaggucucugucuggc.......................................................................   1        0     seq
    ...................uauggaggucucugucuggcu......................................................................   6        0     seq
    ....................auggaggucucugucuggcu......................................................................   2        0     seq
    .........................................................ucugaucguuccccuccAua.................................   5        1     seq
    .........................................................ucugaucguuccccuccAuac................................   1        1     seq
    .........................................................ucugaucguuccccuccAuaca...............................   3        1     seq
```

Provisional ID	:	chr1_199
Score total	:	7.8
Score for star read(s)	:	3.9
Score for read counts	:	5.7
Score for mfe	:	-2.5
Score for randfold	:	-2.2
Score for cons. seed	:	3
Total read count	:	23
Mature read count	:	18
Loop read count	:	0
Star read count	:	5

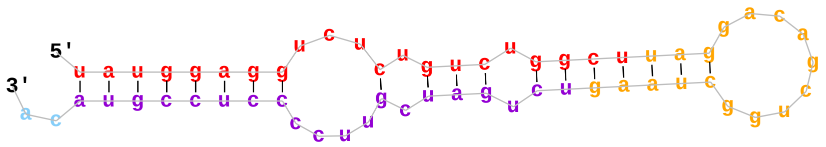

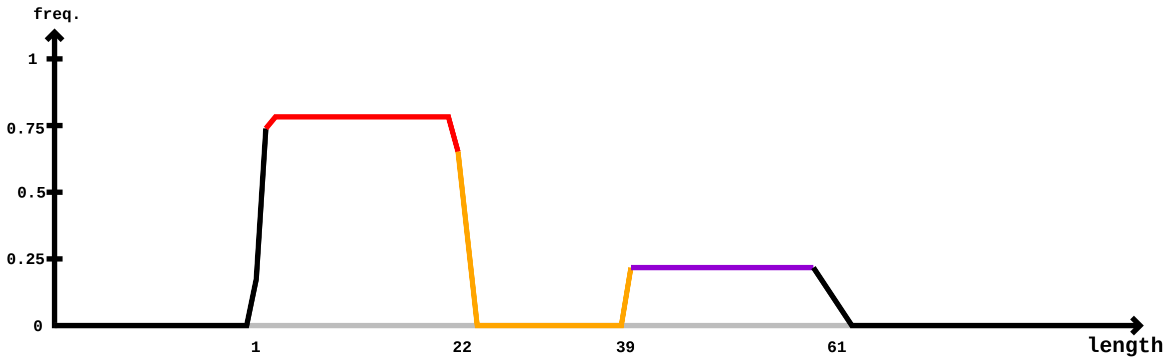


```
                        Mature                          Star
5'- gcagcgauccuacauaaauauauggaggucucugucuggcuuaggacagcuggcuaagucugaucguuccccuccguacagccuuuaaaacugccacucuaaauacuuggu   -3'    obs
    gcagcgauccuacauaaauauauggaggucucugucuggcuuaggacagcuggcuaagucugaucguuccccuccguacagccuuuaaaacugccacucuaaauacuuggu          exp
    ....................((((((((...(.(((.(((((((.........))))))).))).)....))))))))..............((((...........))))   reads      mm        sample
    ...................auauggaggucucugucuggc.......................................................................   1          0         seq
    ...................auauggaggucucugucuggcu......................................................................   3          0         seq
    ....................uauggaggucucugucuggc.......................................................................   2          0         seq
    ....................uauggaggucucugucuggcu......................................................................   11         0         seq
    .....................auggaggucucugucuggcu......................................................................   1          0         seq
    ..........................................................ucugaucguuccccuccgua.................................   2          0         seq
    ..........................................................ucugaucguuccccuccAua.................................   2          1         seq
    ..........................................................ucugaucguuccccuccAuaca...............................   1          1         seq
```

Provisional ID	:	chr15_1658
Score total	:	5.4
Score for star read(s)	:	3.9
Score for read counts	:	-4.9
Score for mfe	:	1.9
Score for randfold	:	1.6
Score for cons. seed	:	3
Total read count	:	2
Mature read count	:	1
Loop read count	:	0
Star read count	:	1

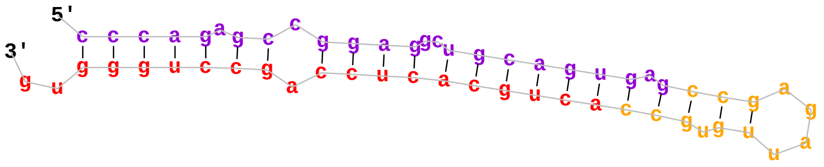

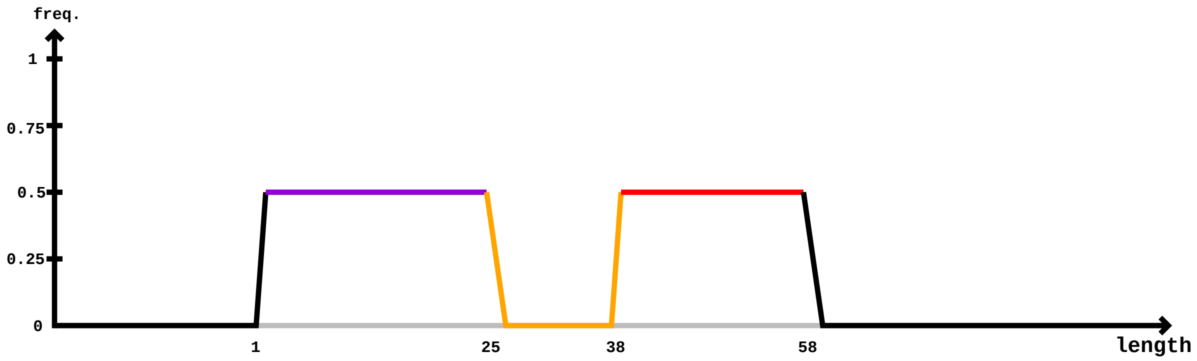


```
                  Star                            Mature
5'- ggugggaggaucgcuugagccccagagccggaggcugcagugagccgagauugugccacugcacuccagccugggugacagagcaagauuuugcuuuaaaacaaaacaaaacaa   -3'    obs
    ggugggaggaucgcuugagccccagagccggaggcugcagugagccgagauugugccacugcacuccagccugggugacagagcaagauuuugcuuuaaaacaaaacaaaacaa          exp
    ...((.((((((((((...((((((.((.((((..(((((((.((((....)).))))))))))))).))))))).)...))))..)))))).))...................    reads      mm         sample
    ....................cccagagccggaggcugcagugag......................................................................    1          0          seq
    .........................................................acugcacuccagUcugggug.....................................    1          1          seq
```

Provisional ID	:	chr2_124
Score total	:	5.9
Score for star read(s)	:	3.9
Score for read counts	:	-4.9
Score for mfe	:	2.4
Score for randfold	:	1.6
Score for cons. seed	:	3
Total read count	:	2
Mature read count	:	1
Loop read count	:	0
Star read count	:	1

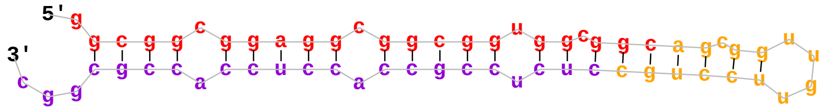

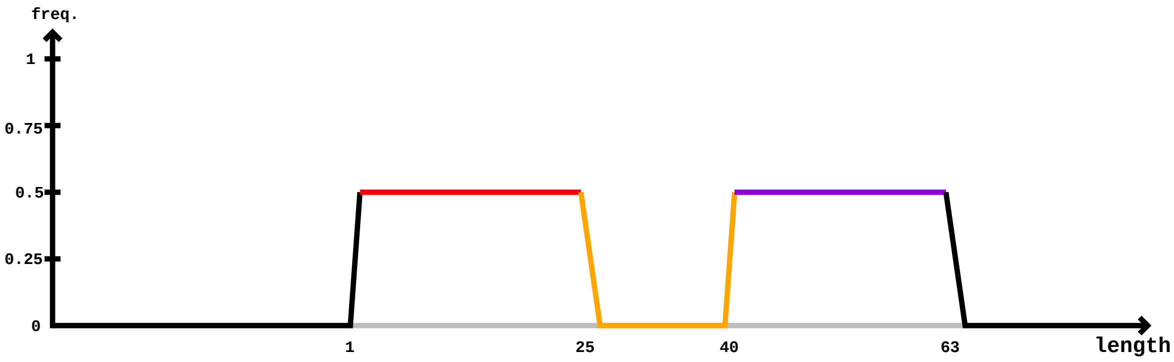


```
                                 Mature                                   Star
5'- cuccgccuccuccucccauccagccugcucggcggcggaggcggcgguggcggcagcgguuguuccugccucuccgccaccuccaccgcggcuuauccccuggcuggcggcg   -3'    obs
    cuccgccuccuccucccauccagccugcucggcggcggaggcggcgguggcggcagcgguuguuccugccucuccgccaccuccaccgcggcuuauccccuggcuggcggcg          exp
    ..(((((....((........((((......((((.(((((.(((((.((.(((((.((....))))))))).))))).))))).))))))))........))..)))))..   reads   mm   sample
    ..............................ggcggcggCggcggcgguggcggc..........................................................   1       1    seq
    .....................................................................cucuccgccaccuccaccgcggU......................   1       1    seq
```

Provisional ID	:	chr6_387
Score total	:	5.2
Score for star read(s)	:	-1.3
Score for read counts	:	0
Score for mfe	:	1.9
Score for randfold	:	1.6
Score for cons. seed	:	3
Total read count	:	14
Mature read count	:	14
Loop read count	:	0
Star read count	:	0

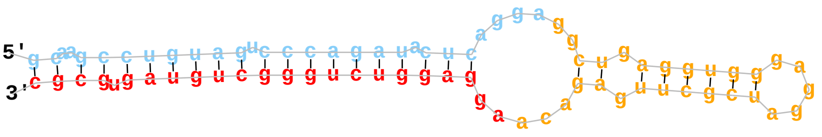

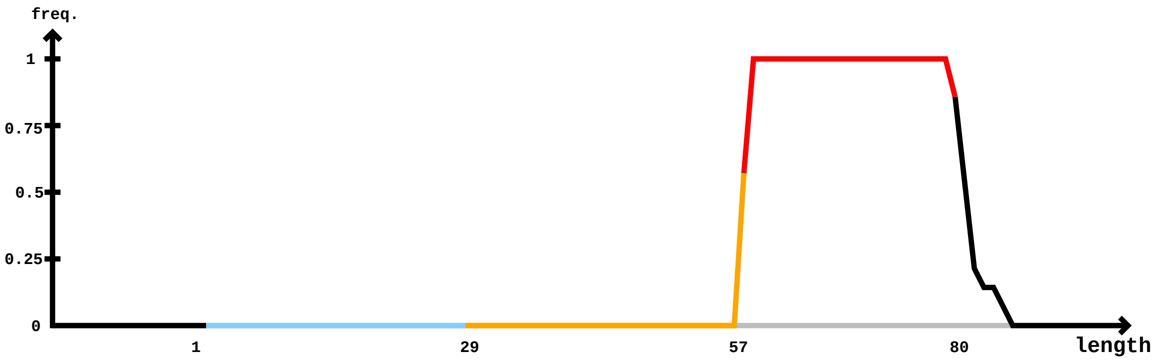

Star Mature

```
5'- cugggugugguggugcaagccuguagucccagauacucaggaggcugaggugggaggaucgcuugagacaaggaggucugggcuguagugcgcuaugccaaucagugucu   -3'   exp
    ((((.((..((((((((...((((((.(((((((.(((......((.((((((.....)))))).))).....))))))))))))))))))))))))..)).)))).....   reads   mm   sample
    ......................................................................aggagUucugggcuguagugcg..................   2   1   seq
    ......................................................................aggagUucugggcuguagugcgc.................   5   1   seq
    ......................................................................aggagUucugggcuguagugcgcu................   1   1   seq
    .......................................................................ggagUucugggcuguagugcgc.................   1   1   seq
    .......................................................................ggagUucugggcuguagugcgcu................   2   1   seq
    .......................................................................ggagUucugggcuguagugcgcua...............   1   1   seq
    .......................................................................ggagUucugggcuguagugcgcuaug.............   1   1   seq
    .......................................................................ggagUucugggcuguagugcgcuaugc............   1   1   seq
```
